# Supplementary material for: Role of miR-944/MMP10/AXL- axis in lymph node metastasis in tongue cancer
Source: Commun Biol. 2023 Jan 17;6:57. doi: 10.1038/s42003-023-04437-6 (PMC9845355; doi:10.1038/s42003-023-04437-6)
Supplement: Supplementary file 2 — Supplementary Information [file 42003_2023_4437_MOESM2_ESM.pdf]

Supplementary Information for

Role of *miR-944/MMP10/AXL*- axis in Lymph Node Metastasis in Tongue Cancer

Bhasker Dharavath[1,2], Ashwin Butle[1], Ankita Pal[1], Sanket Desai[1,2], Pawan Upadhyay[1,2], Aishwarya Rane[1], Risha Khandelwal[1], Sujith Manavalan[1], Rahul Thorat[3], Kavita Sonawane[4], Richa Vaish[2,4], Poonam Gera[2,5], Munita Bal[2,6], Anil K. D'Cruz[4,7], Sudhir Nair[2,4*], Amit Dutt[1,2*]

[1]Integrated Cancer Genomics Laboratory, Advanced Centre for Treatment, Research, and Education in Cancer, Kharghar, Navi Mumbai, Maharashtra, 410210, India
[2]Homi Bhabha National Institute, Training School Complex, Anushakti Nagar, Mumbai, Maharashtra, 400094, India
[3]Laboratory Animal Facility, Advanced Centre for Treatment, Research and Education in Cancer, Kharghar, Navi Mumbai, Maharashtra, 410210, India
[4]Division of Head and Neck Oncology, Department of Surgical Oncology, Tata Memorial Hospital, Tata Memorial Centre, Parel, Mumbai, 400012, India
[5]Tissue Biorepository, Advanced Centre for Treatment Research and Education in Cancer, Kharghar, Navi Mumbai, Maharashtra, 410210, India
[6]Department of Pathology, Tata Memorial Hospital, Tata Memorial Centre, Parel, Mumbai, 400012, India
[7]Apollo Cancer Center, Apollo Hospitals, CBD Belapur, Navi Mumbai, 400614, India

*Corresponding author:
Dr. Sudhir Nair (sudhirvr@gmail.com), or
Dr. Amit Dutt (adutt@actrec.gov.in)

Supplementary Figure 1 to 25 and Table 1 to 8

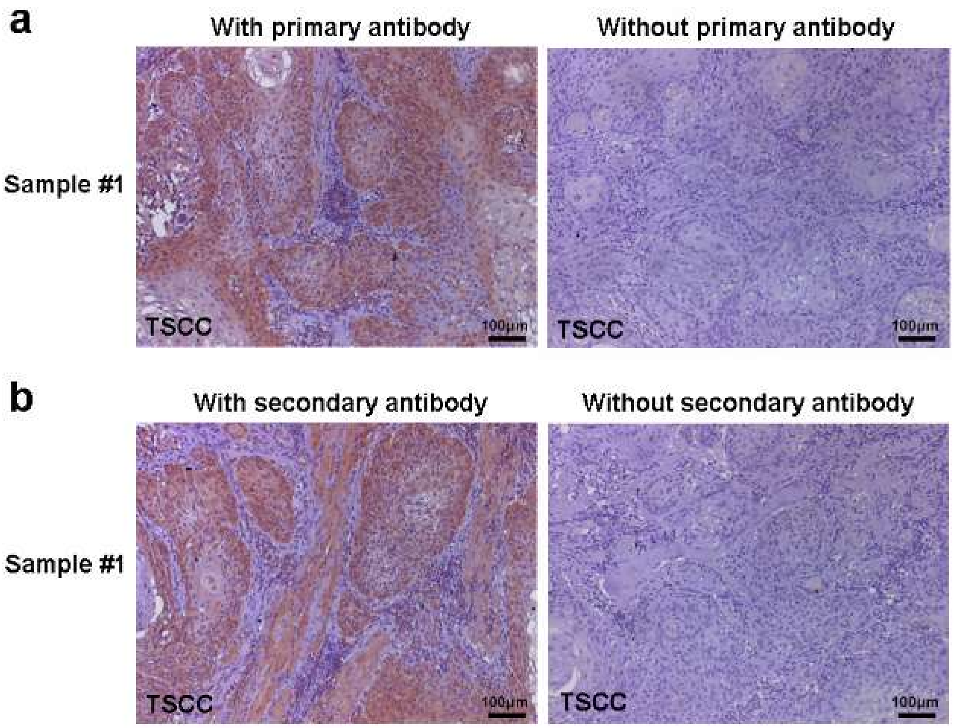

c

Scoring Grade	MMP10 (n=98)	
	Node Negative (n=85), n(%)	Node positive (n=13), n(%)
Strong staining	10 (12%)	5 (38%)
Moderate staining	25 (29%)	5 (38%)
Weak staining	36 (42%)	3 (23%)
No staining	14 (16%)	0 (0%)
p-value	0.0035	

Supplementary Figure 1: Immunohistochemistry of MMP10 in N-zero clinical trial samples. Representative IHC stained images of tongue tumors are shown with a scale bar (100 μm). The brown color indicates positive staining for MMP10 protein. To demonstrate the MMP10 antibody specificity, immunohistochemical staining was performed (a) with and without primary antibody; and (b) with and without secondary antibody, as negative controls. (c) Tabular representation for quantification of MMP10 immunostaining data (n = 98). Statistical difference in IHC scores between node-positive and node-negative tumor samples are estimated using Student's unpaired t-test and $p<0.05$ is considered as the threshold for significance.

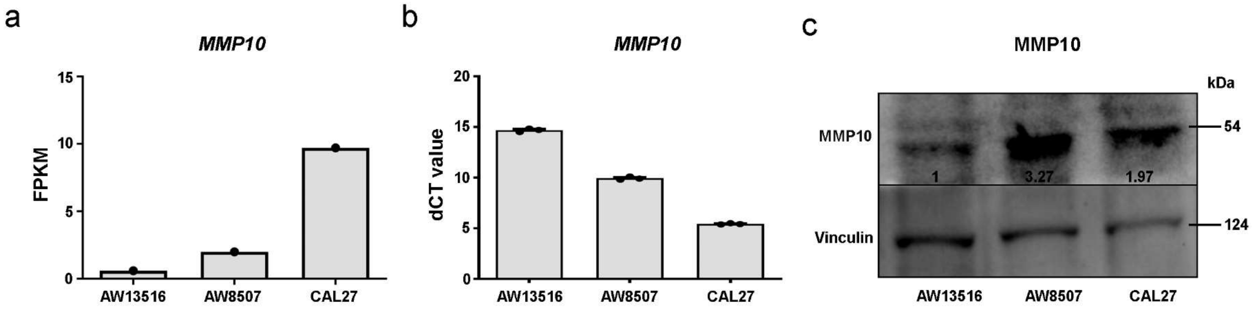

Supplementary Figure 2: Expression of MMP10 in tongue cancer cell lines. (a) Transcriptome sequencing data was used for estimation of *MMP10* transcript in the cell lines. The bar plot depicts the *MMP10* expression in Fragments Per Kilobase of transcript per Million mapped (FPKM) values. (b) qRT-PCR analysis to quantify expression of *MMP10* in the cell lines. *GAPDH* was used as an internal control. Real-time PCR data is plotted as a bar plot representation of dCT values of *MMP10* in tongue cancer cell lines. Data are shown as means ± SD. (c) MMP10 expression at the protein level in tongue cancer cell lines was measured by Western blotting. Vinculin was used as loading control. Numbers on the blot indicate intensity ratio for MMP10, normalized to Vinculin levels in the respective cell lines. Data shown are representative of n = 3 independent experiments.

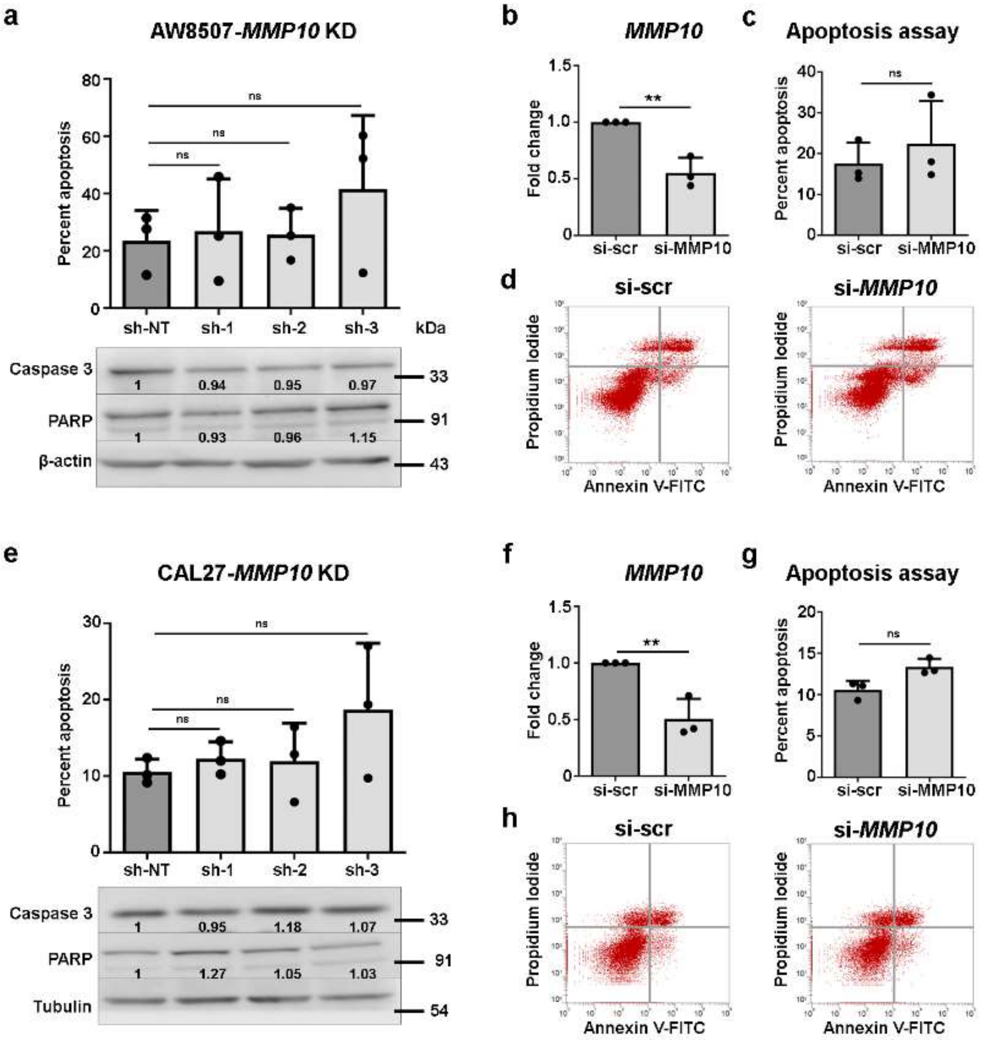

Supplementary Figure 3: Knockdown of *MMP10* does not affect apoptosis of tongue cancer cells. (a, e) Bar plot of cell viability assay performed using propidium iodide (PI) staining indicates the percentage of late apoptotic and necrotic cells in *MMP10* knockdown clones (sh-1, sh-2 and sh-3) and non-targeting shRNA control clones (sh-NT) of AW8507 (a) and CAL27 (e). Western blots indicate the expression of Caspase 3 and PARP cleavage in the *MMP10* knockdown and vector control cells. Numbers on the blot indicate intensity ratio of target protein expression with respect to the vector control lane. β-actin or Tubulin was used as loading control. (b, f) qRT-PCR analysis indicating siRNA mediated knockdown of *MMP10* in AW8507 (b) and CAL27 (f) cells. GAPDH was used for normalization. (c, g) Bar plot of Annexin V-FITC/PI staining indicating percentage of early- and late apoptotic cells in *MMP10*-knockdown and scrambled control clones of AW8507 (c) and CAL27 (g). (d, h) Representative scatter plots depicting PI (y-axis) vs. annexin V-FITC (x-axis) staining in *MMP10* knockdown and scrambled control of AW8507 (d) and CAL27 (h). In each scatterplot, top-left quadrant indicates necrotic cells, top-right quadrant indicates late apoptotic cells, bottom-left quadrant indicates live cells and bottom-right quadrant indicates early apoptotic cells. Data are shown as means ± SD. *p*-values are from Student's unpaired t-test and denoted as *ns* (not significant); **, $p<0.01$. Data shown are representative of n = 3 independent experiments.

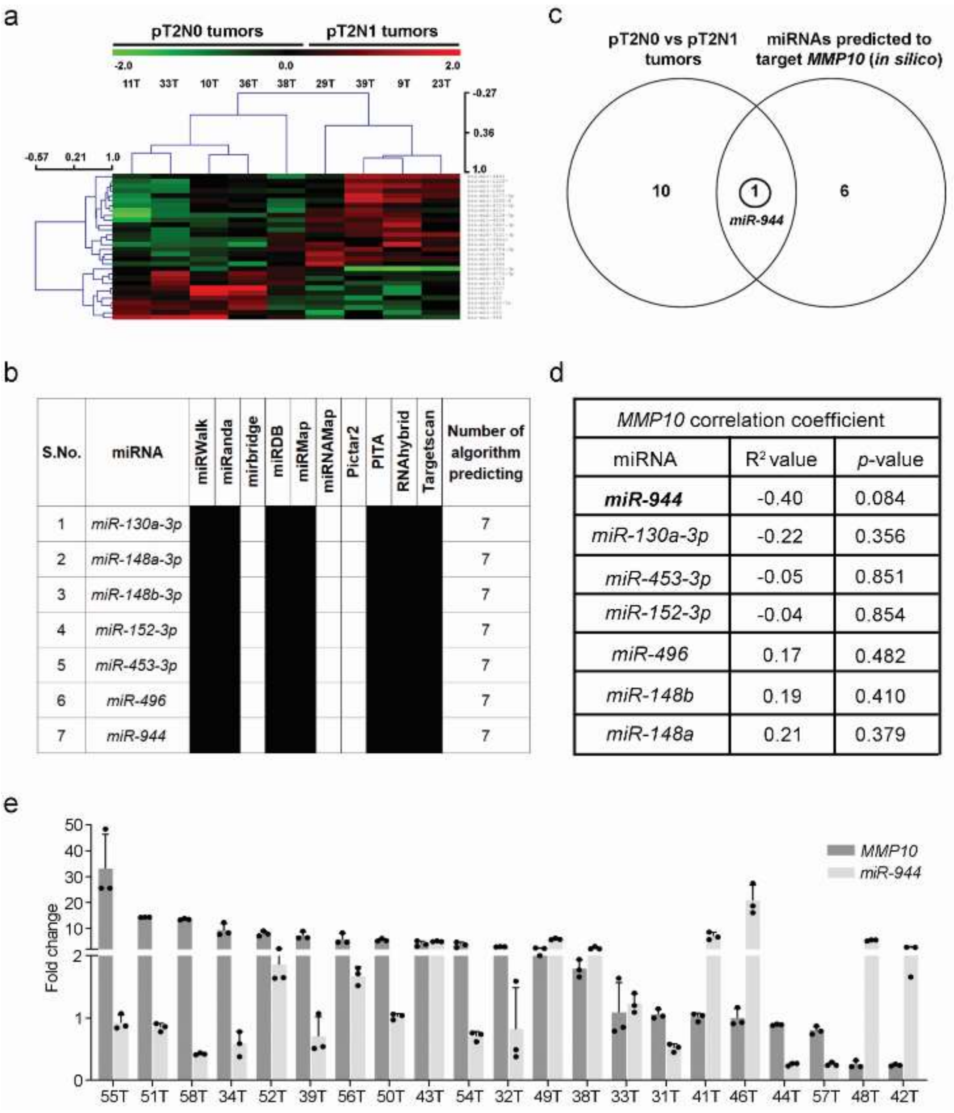

Supplementary Figure 4: Identification and real-time PCR based validation of miRNAs targeting *MMP10* 3'-UTR. (a) Heatmap representation of 33 differentially expressed miRNAs (-0.5<fold change>0.5; *p*-value<0.05) between pT2N0 and pT2N1 primary tumors. Of the 33 deregulated miRNAs, 22 miRNAs were upregulated and 11 miRNAs were downregulated. Red and green color in the heatmap denotes upregulated and downregulated miRNAs, respectively. (b) Tabular representation of *in silico* analysis performed to identify miRNAs predicted to target 3'-UTR of *MMP10* using 10 different miRNA binding site prediction tools. miRNAs predicted by 7 of the 10 tools are represented in the table. (c) Venn diagram indicating the overlapping miRNA between downregulated miRNAs identified from pT2N0 vs pT2N1 tumors and miRNAs predicted to be targeting *MMP10* by 7 out of 10 tools. (d) Real-time PCR based tabular representation of miRNAs ranked based on Pearson correlation values for delta-delta Ct of miRNAs predicted to be targeting *MMP10* and *MMP10* transcript in 20 primary tumor samples along with paired adjacent normal. *miR-944* showing the highest negative correlation is highlighted in bold. (e) The bar plot representation of *MMP10* and *miR-944* relative fold change in tongue tumor and normal paired samples (n = 20). The data was normalized against internal reference control for *MMP10* and miRNAs with *GAPDH* and *U6*, respectively. Relative fold change was obtained by comparing the expression in the corresponding paired normal sample for each patient. The x-axis shows the patient sample IDs. Data are shown as means ± SD. Data shown in (d, e) are representative of n = 3 independent experiments.

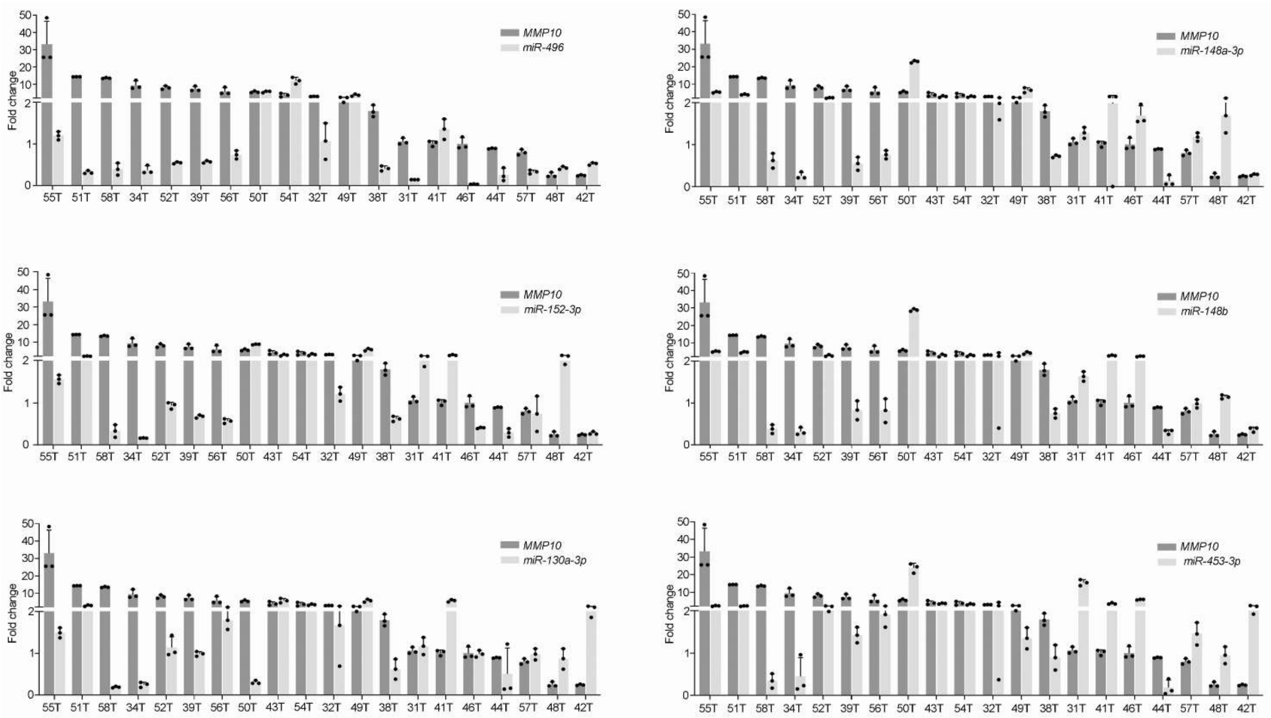

Supplementary Figure 5: qRT-PCR analysis of miRNAs targeting 3'-UTR of *MMP10*. The bar plot representation of *MMP10* and six miRNA relative fold change in tongue tumor and paired normal samples (n = 20). The data was normalized against internal reference control for *MMP10* and miRNAs with *GAPDH* and *U6*, respectively. Relative fold change was obtained by comparing the expression in the corresponding paired normal sample for each patient. The x-axis shows the patient sample IDs. Data are shown as means ± SD. Data shown are representative of n = 3 independent experiments.

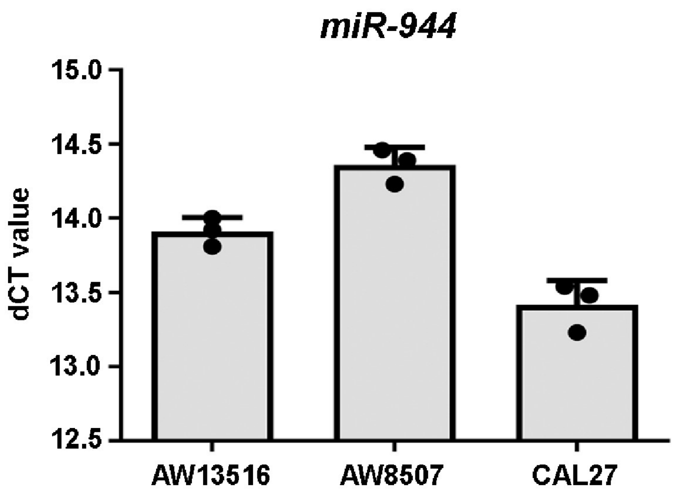

Supplementary Figure 6: Expression of *miR-944* in tongue cancer cell lines. qRT-PCR analysis for estimation of *miR-944* expression in tongue cancer cell lines. Expression of *miR-944* was normalized with *U6*. Data are shown as means ± SD. Data shown are representative of n = 3 independent experiments.

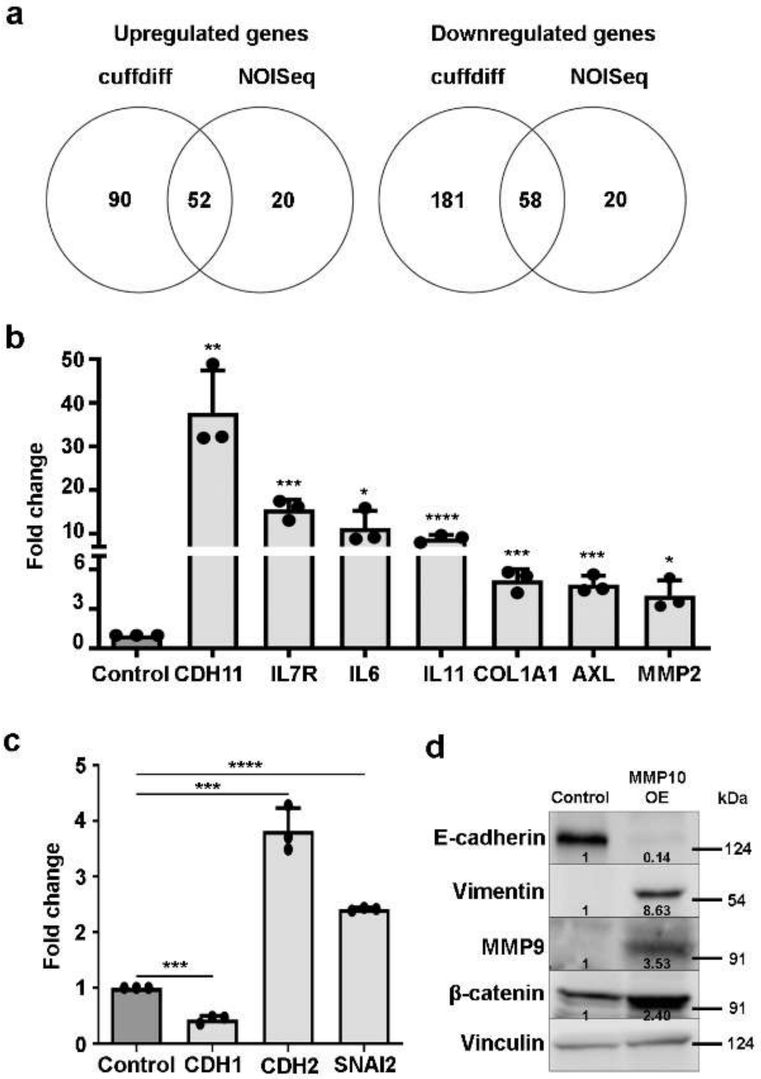

Supplementary Figure 7: Differential expression analysis and validation of deregulated genes and EMT markers upon overexpression of *MMP10* in AW13516 cell line. (a) Venn diagram of differentially expressed genes (DEGs) in AW13516 cells overexpressing empty vector or *MMP10*. Venn diagrams indicate the number of downregulated and upregulated genes (-1.5<log2FC>1.5; $p < 0.05$) identified by cuffdiff and NOISeq tools. The intersection in the Venn diagram circles represents the DEGs commonly identified by both tools. (b) qRT-PCR validation of genes identified from transcriptome sequencing data of AW13516 cells overexpressing *MMP10*. Bar plot depicts the expression of deregulated genes upon overexpression of *MMP10* compared to vector control cells. (c) Bar plot showing the qRT-PCR validation data of EMT marker genes deregulated upon overexpression of *MMP10*. *GAPDH* was used for normalization. (d) Western blot analysis of E-cadherin, Vimentin, MMP9 and β-catenin in AW13516 cells overexpressing *MMP10* or empty vector. Numbers on the blot indicate intensity ratio of target protein expression in *MMP10* overexpression clones compared to the vector control lane. Vinculin was used as reference control. Data are shown as means ± SD. *p*-values were calculated using Student's unpaired t-test and denoted as *, $p<0.05$; **, $p<0.01$; ***, $p<0.001$; ****, $p<0.0001$. Data shown in (b-d) are representative of n = 3 independent replicates.

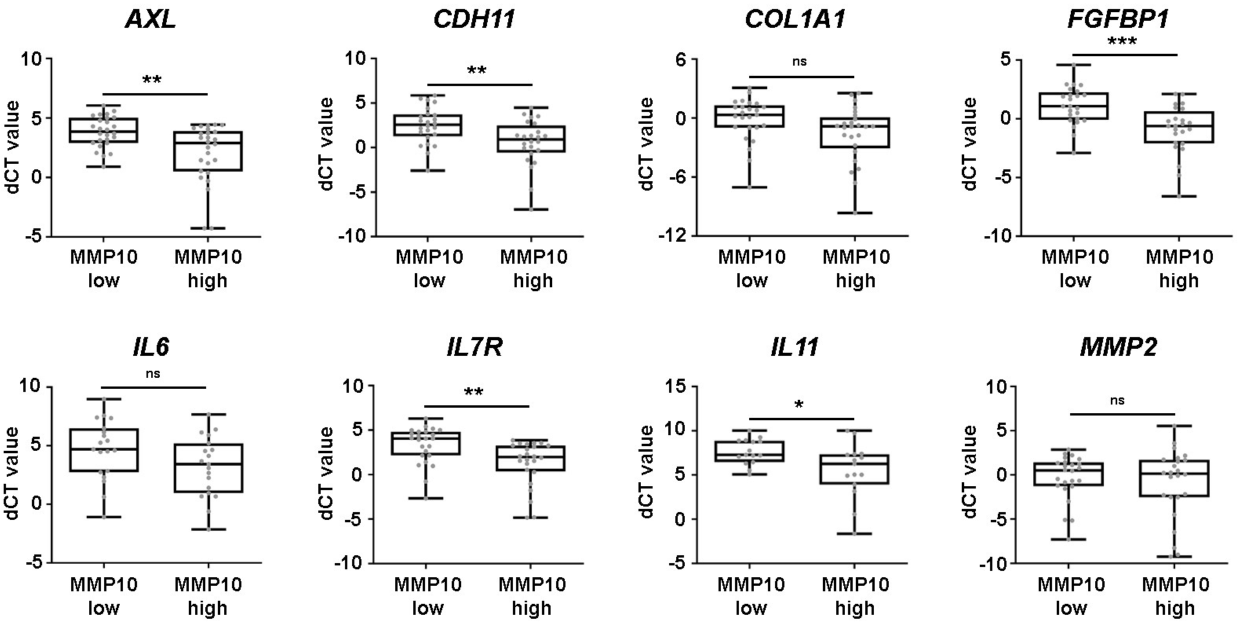

Supplementary Figure 8: Validation of metastasis pathway-related genes and correlation with *MMP10* expression in tongue tumor samples. qRT-PCR analysis of *AXL*, *CDH11*, *COL1A1*, *FGFBP1*, *IL6*, *IL7R*, *IL11*, and *MMP2* transcript expression in primary tongue tumor samples (n = 52). Boxplots represent the dCT values of genes and their significance between tumor samples with low (*MMP10* low) and high (*MMP10* high) expression of *MMP10* (based on median expression). *GAPDH* was used for normalization. The middle line in the boxplot shows median along with the lower (Q1) and upper quartiles (Q3) as boxes. The whiskers represent the minimum and maximum values. Data are shown as means ± SD. *p*-value was calculated using Student's unpaired t-test and denoted as *ns* (not significant); *, $p<0.05$; **, $p<0.01$; ***, $p<0.001$. Data shown are representative of n = 3 independent replicates of real-time PCR data for each sample.

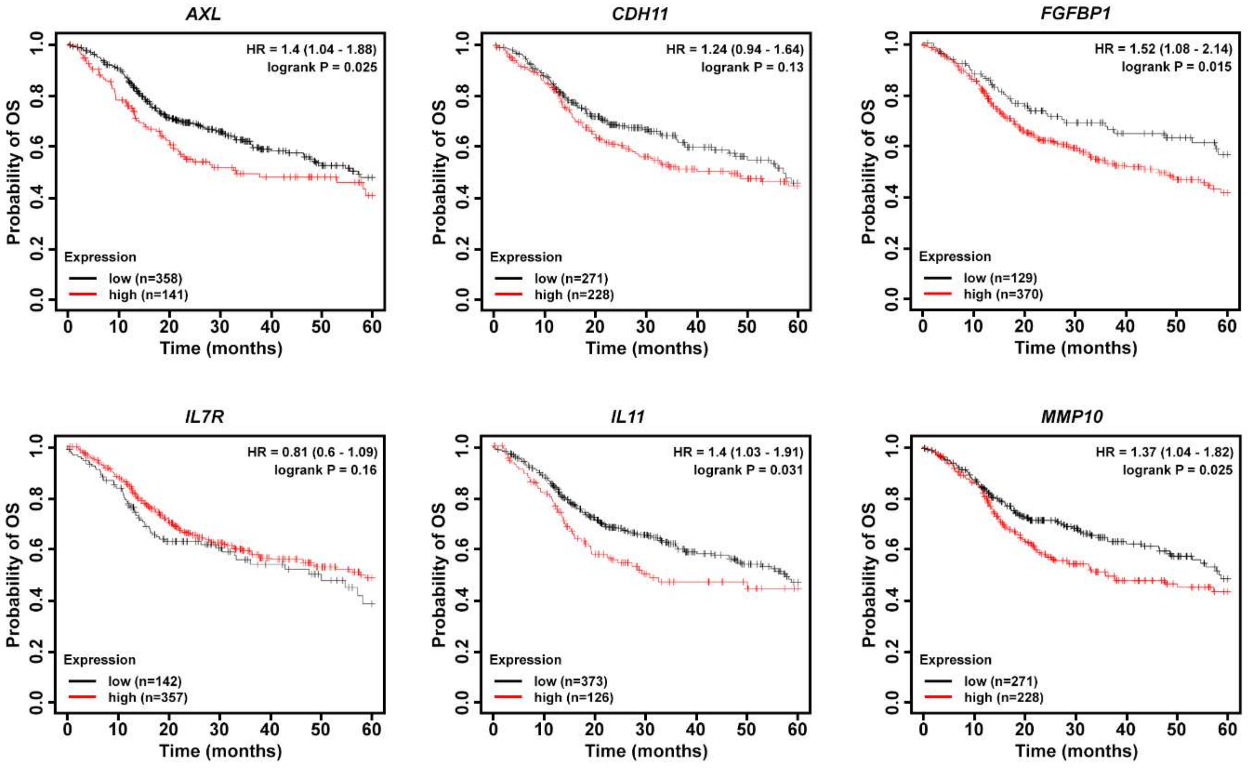

Supplementary Figure 9: Kaplan-Meier survival curves for overall survival in TCGA-HNSC data. Kaplan-Meier (KM) survival curves for overall survival (OS) of TCGA-HNSC data based on the expression of *AXL*, *CDH11*, *FGFBP1*, *IL7R*, *IL11* and *MMP10*. The red and black lines denote the high and low expression of the genes in the KM plots. The number of samples in each group is denoted. The log-rank test was used to determine the statistical differences in median survival and $p<0.05$ was considered as statistically significant.

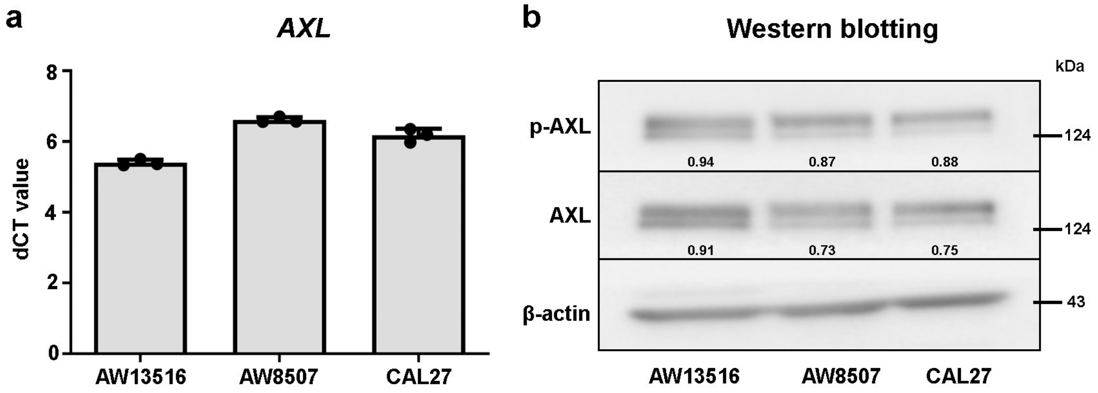

Supplementary Figure 10: Expression of AXL transcript and protein in tongue cancer cell lines. (a) qRT-PCR analysis for estimation of *AXL* in tongue cancer cell lines. Expression of *AXL* was normalized with *GAPDH*. Data are shown as means ± SD. (b) Immunoblot showing expression of p-AXL, AXL and β-actin protein in tongue cancer cell lines. Numbers on the blot indicate intensity ratio for p-AXL and AXL, normalized to β-actin levels in the respective cell lines. Data shown are representative of n = 3 independent experiments.

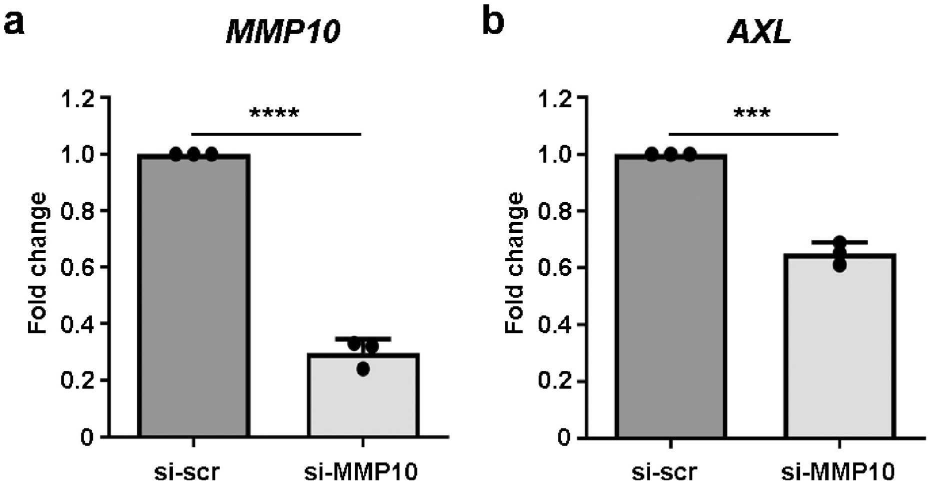

Supplementary Figure 11: siRNA-mediated knockdown of *MMP10* downregulates *AXL* in AW13516-*MMP10* overexpressing cells. (a, b) qRT-PCR analysis indicating the expression of *MMP10* (a) and *AXL* (b) in AW13516-*MMP10* cells with siRNA-mediated knockdown of *MMP10* or scrambled control. Expression of genes were normalized with *GAPDH*. Data are shown as means ± SD. *p*-values were calculated using Student's unpaired t-test and denoted as ***, $p<0.001$; ****, $p<0.0001$. Data shown are representative of n = 3 independent experiments.

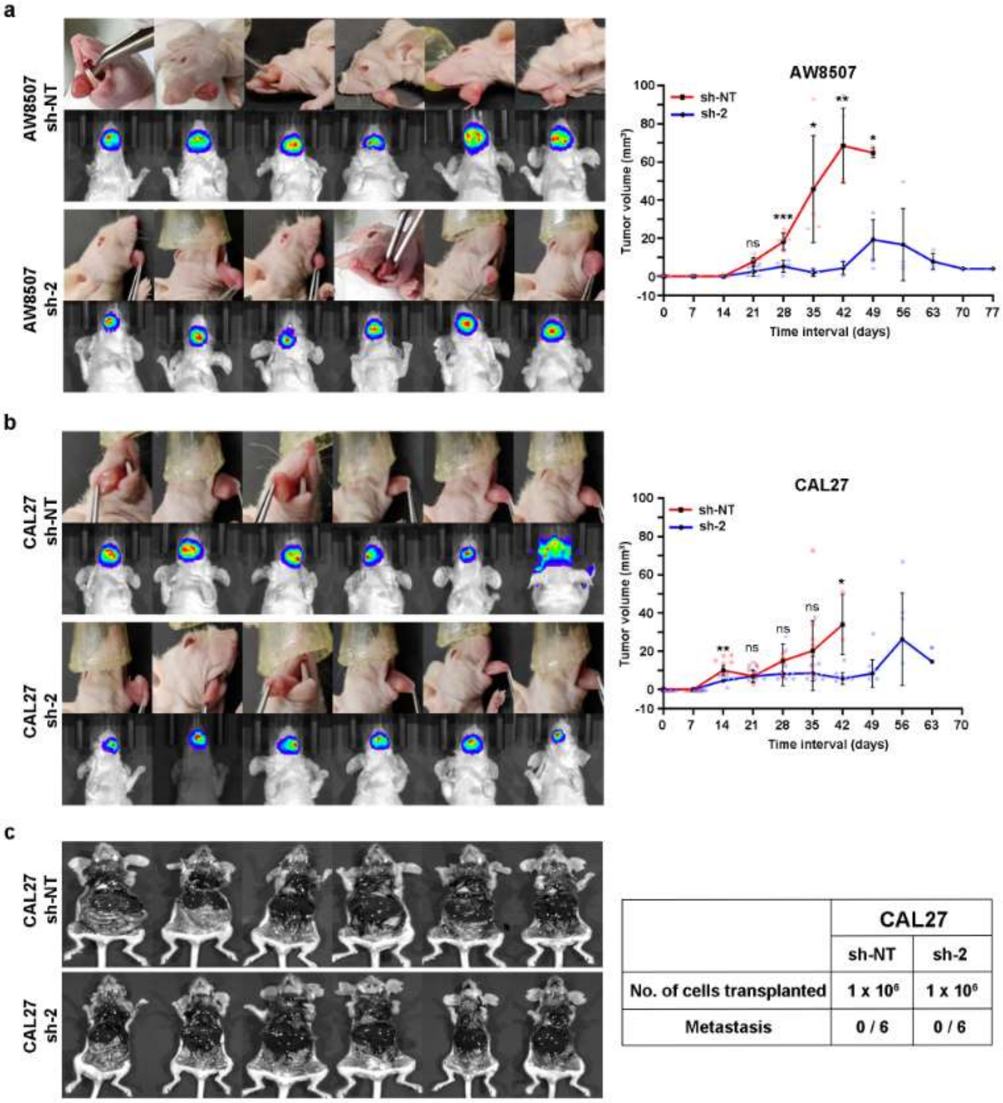

	CAL27	
	sh-NT	sh-2
No. of cells transplanted	1 x 10^6	1 x 10^6
Metastasis	0 / 6	0 / 6

Supplementary Figure 12: Knockdown of *MMP10* suppresses tumorigenesis of tongue cancer *in vivo*. (a) Orthotopic tongue tumor growth in mice injected with luciferase-labeled AW8507 clones of *MMP10* knockdown (sh-2) or non-targeting control (sh-NT). 2x10^6 cells were injected orthotopically into the tongue of the 6-8 week old nude mice and imaged by an *In Vivo* live Imaging System (IVIS) at regular intervals of 7 days starting at day 7 to day 77. Graph shows the caliper measurements of tumor volume at 7-day time intervals, plotted as means ± SD (n = 6/group). (b) Orthotopic tongue tumors in mice injected with luciferase-labelled CAL27 clones of *MMP10* knockdown (sh-2) or sh-NT control cells (1x10^6 cells were injected). Graph shows caliper measurements of tumor volume, plotted as means ± SD (n = 6/group). (c) IVIS imaging for detection of metastasis in mice injected with CAL27 clones of *MMP10* knockdown or non-targeting control (n = 6/group). Bioluminescence imaging was performed after the resection of primary tongue tumors in the mice. IVIS imaging of mice did not show regional or distant metastasis. Table shows the number of cells injected orthotopically into the tongue of the mice and number of mice with metastasis in lymph nodes or distant organs. *p*-value is denoted as *ns* (not significant); *, *p*<0.05; **, *p*<0.01; ***, *p*<0.001.

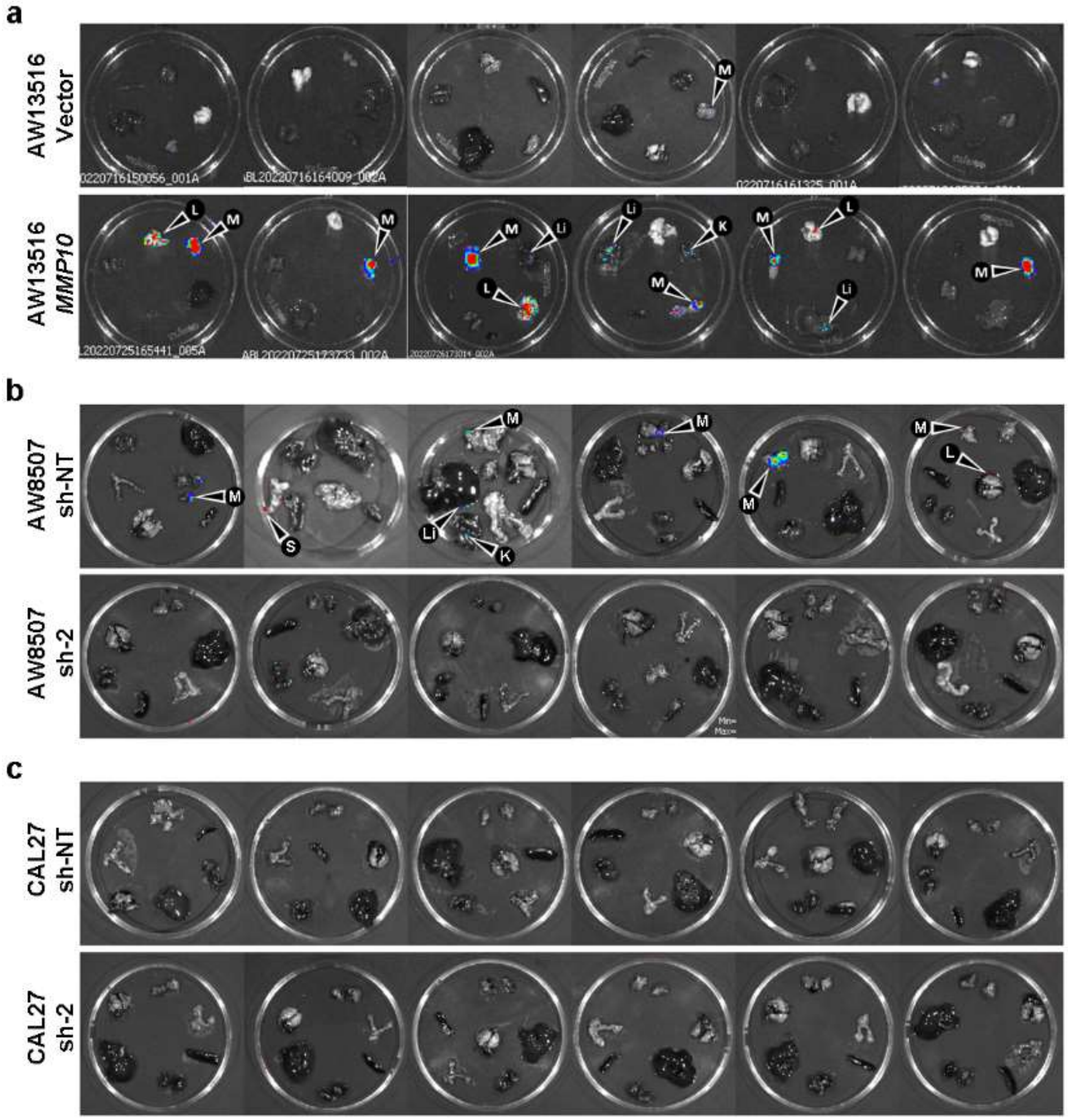

Supplementary Figure 13: IVIS imaging of mice organs showing metastasis *in vivo*. IVIS imaging of mice internal organs (sub-maxillary glands and cervical lymph nodes (M), lungs (L), liver (Li), kidney (K), spleen (S), uterus (U) and ovaries (O)) after necropsy to detect metastasis in mice injected with (a) AW13516-vector control or *MMP10*-overexpression clones, (b) AW8507-vector control or *MMP10*-knockdown clones, and (c) CAL27-vector control or *MMP10*-knockdown clones. Bioluminescence signal from the organs indicate metastasis.

Supplementary Figure 14 to 25: Uncropped Western blots

These uncropped Western blots are presented as the source data of:

1) Figure 2a, d, h
2) Figure 4a, b
3) Supplementary Figure 2c
4) Supplementary Figure 3a, e
5) Supplementary Figure 7d
6) Supplementary Figure 10b

Supplementary Figure 14: Uncropped Western blots used in Figure 2a (AW13516-*MMP10* overexpression). Cropped sections marked on the blots are used as figures in the manuscript:

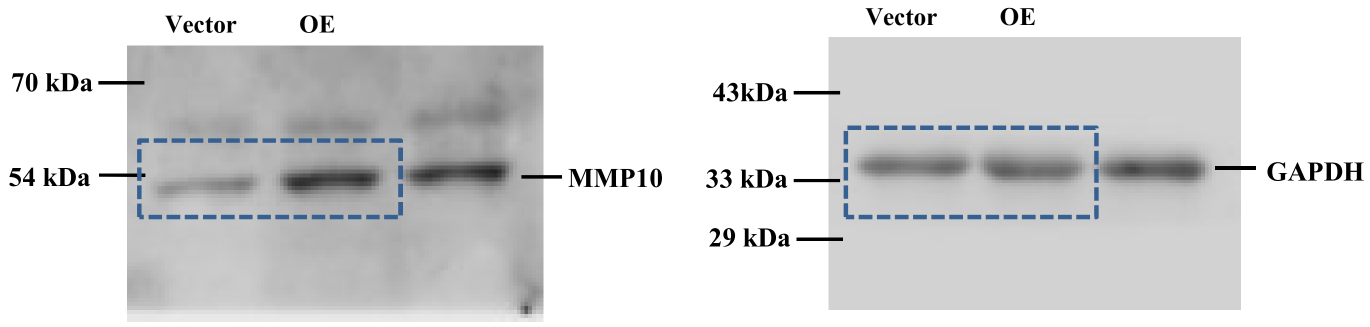

Supplementary Figure 15: Uncropped Western blots used in Figure 2d (AW8507-*MMP10* knockdown) and Figure 2h (CAL27-*MMP10* knockdown). Cropped sections marked on the blots are used as figures in the manuscript:

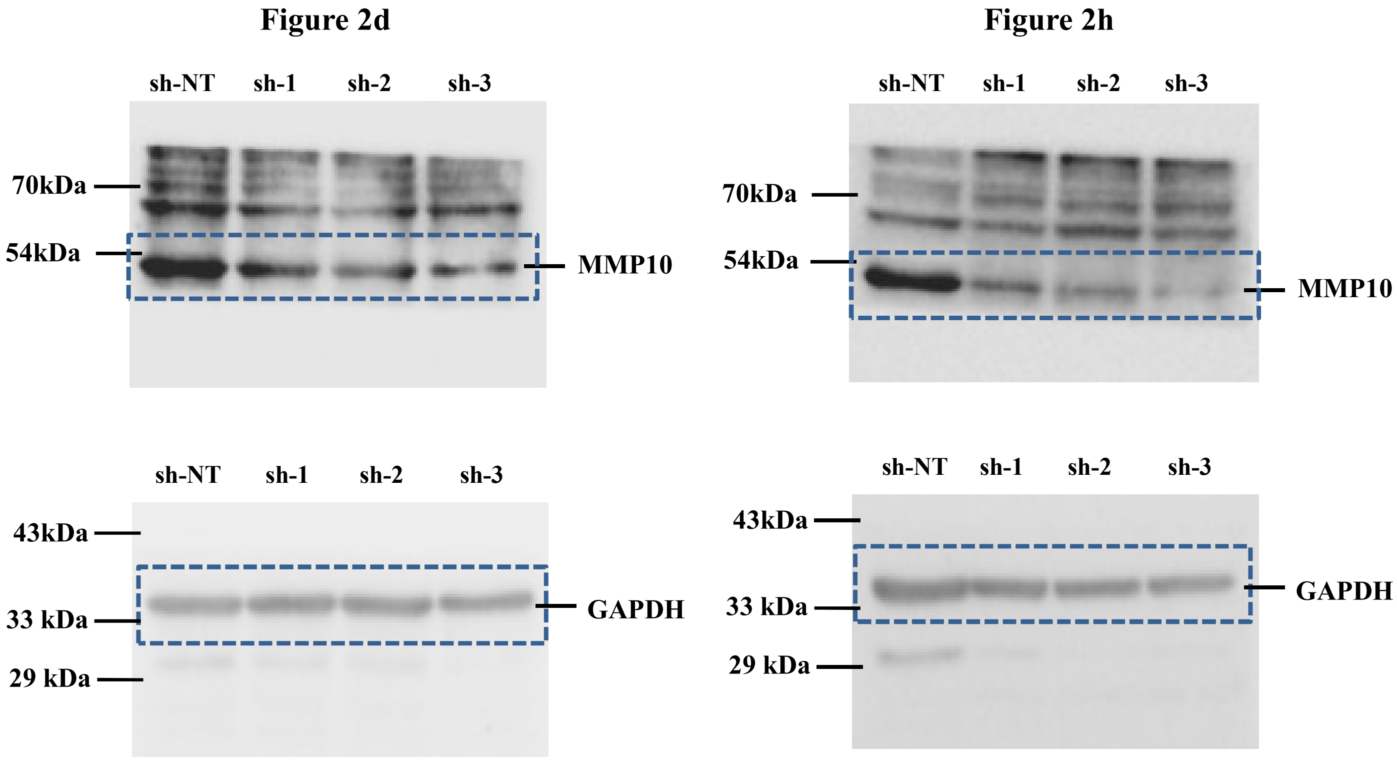

Supplementary Figure 16: Uncropped Western blots used in Figure 4a (column 1—AW13516-*MMP10* overexpression). Cropped sections marked on the blots are used as figures in the manuscript:

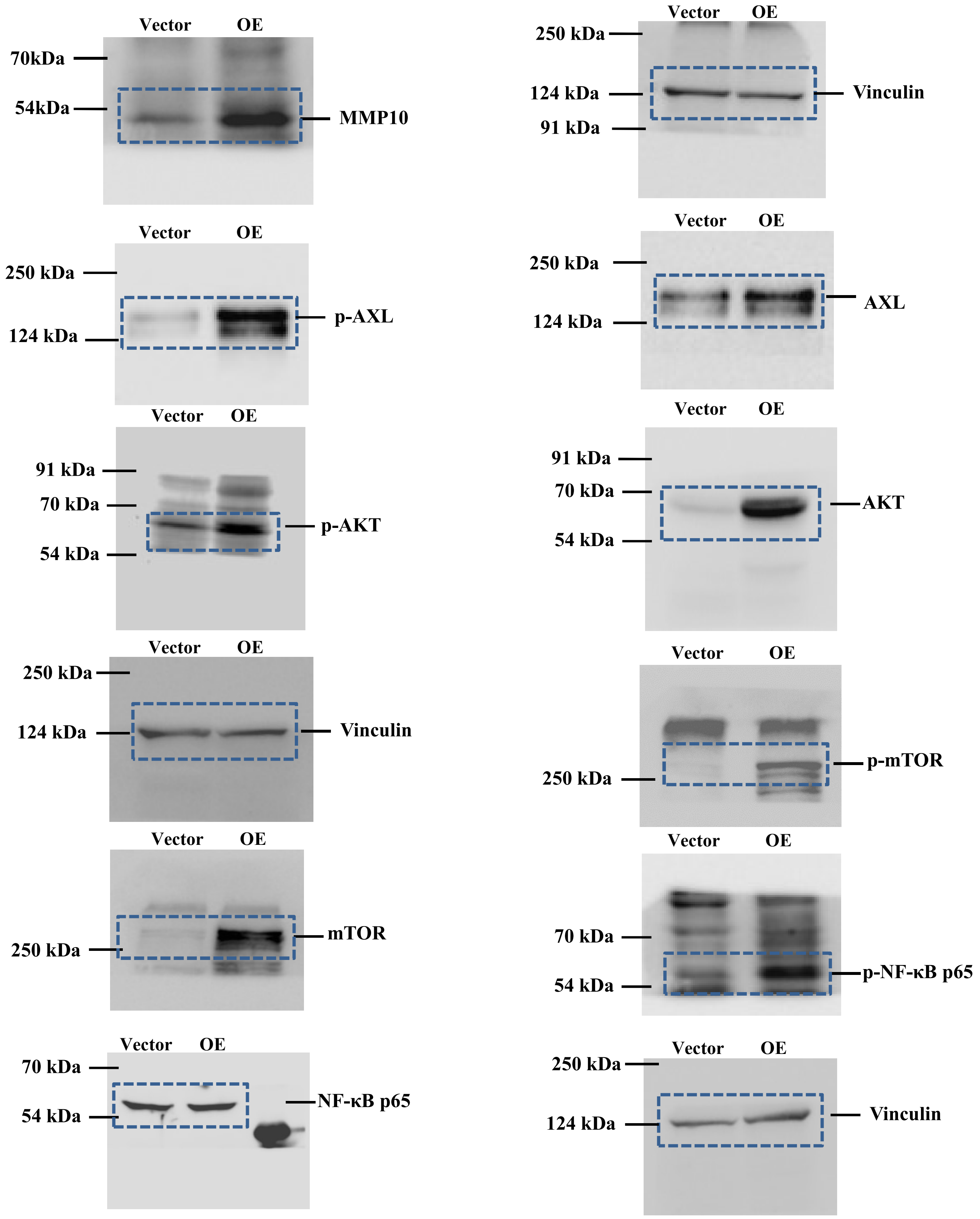

Supplementary Figure 17: Uncropped Western blots used in Figure 4a (column 2—AW8507-*MMP10* knockdown). Cropped sections marked on the blots are used as figures in the manuscript:

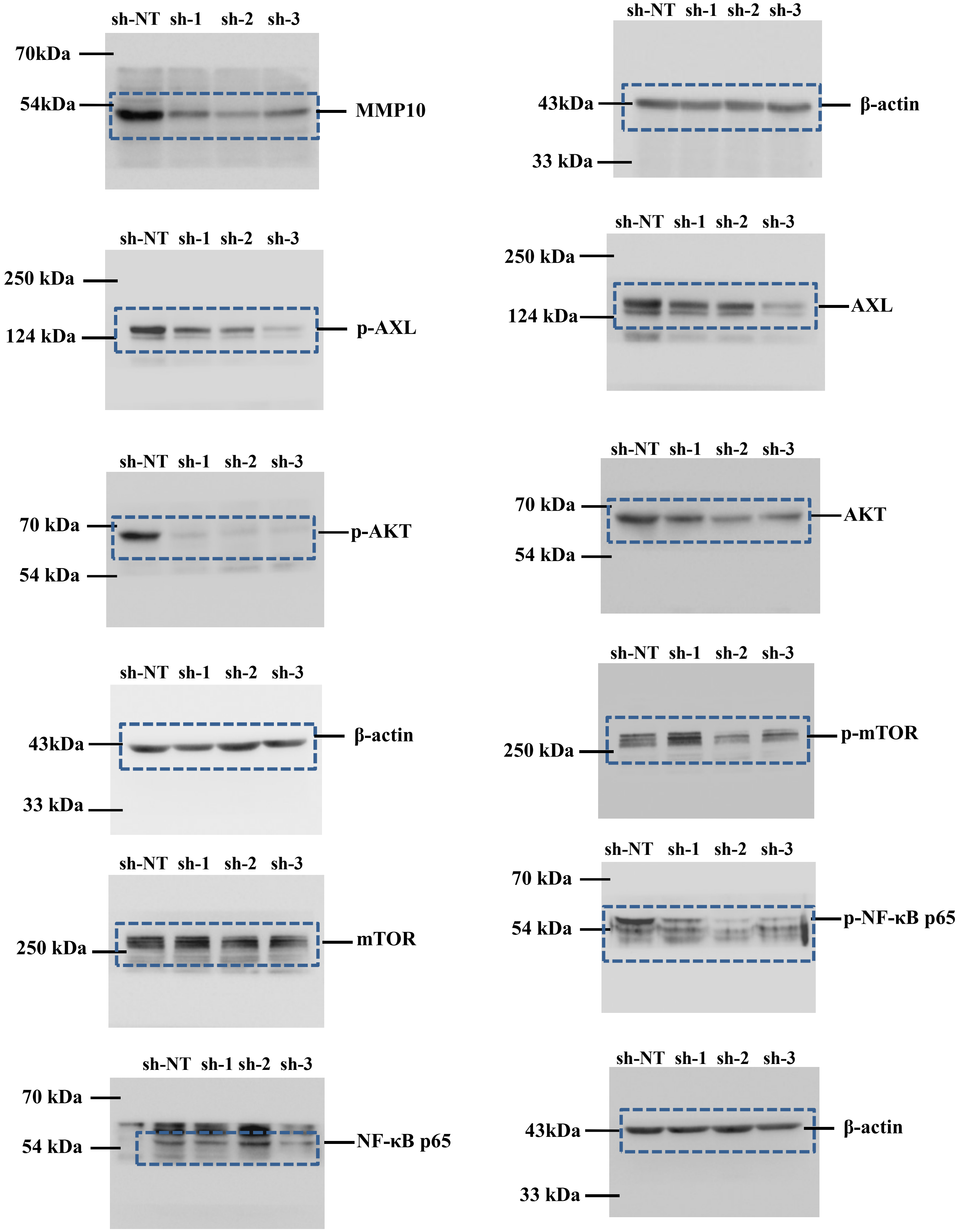

Supplementary Figure 18: Uncropped Western blots used in Figure 4a (column 3—CAL27-*MMP10* knockdown). Cropped sections marked on the blots are used as figures in the manuscript:

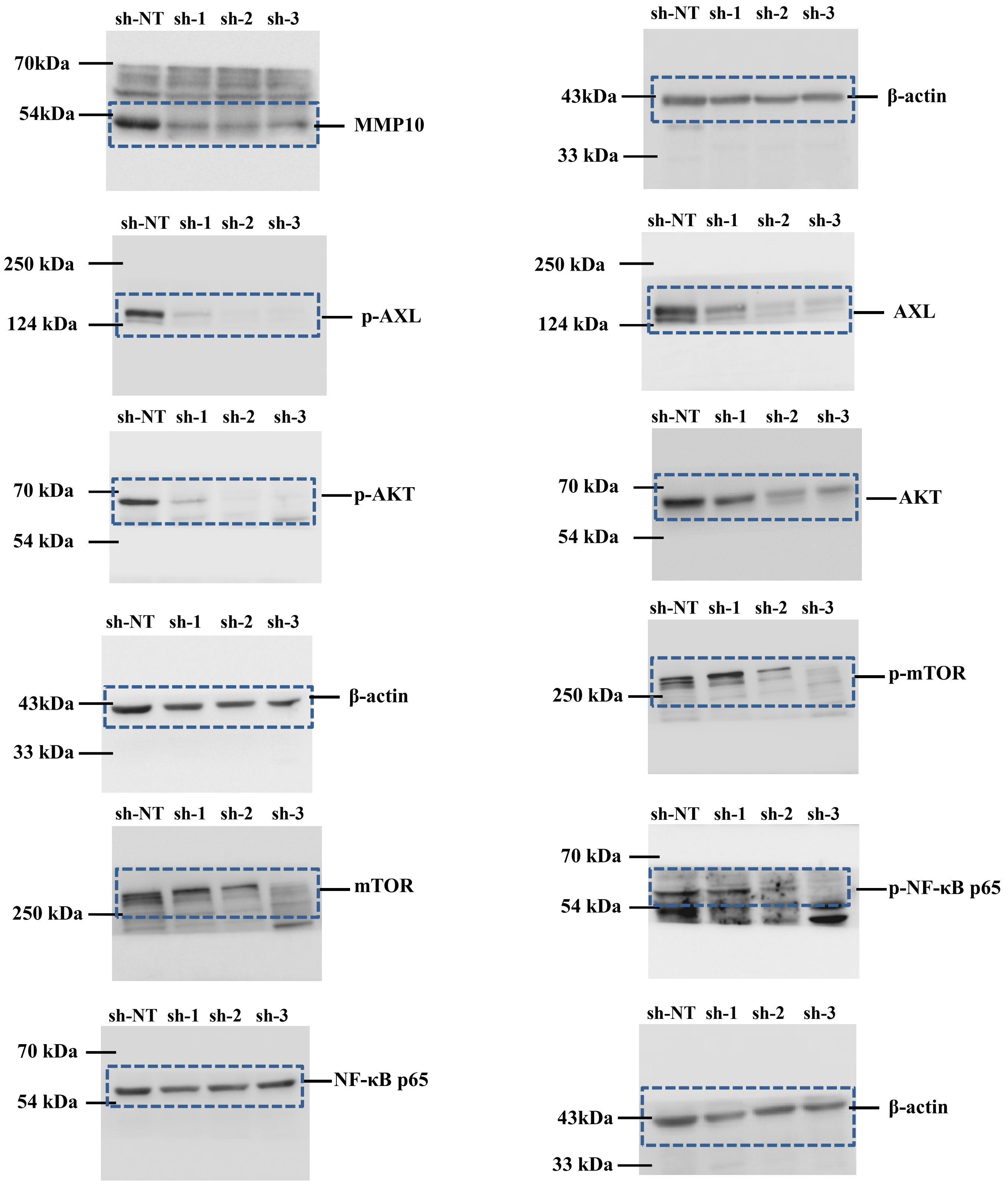

Supplementary Figure 19: Uncropped Western blots used in Figure 4b (column 1—AW13516-*AXL* overexpression). Cropped sections marked on the blots are used as figures in the manuscript:

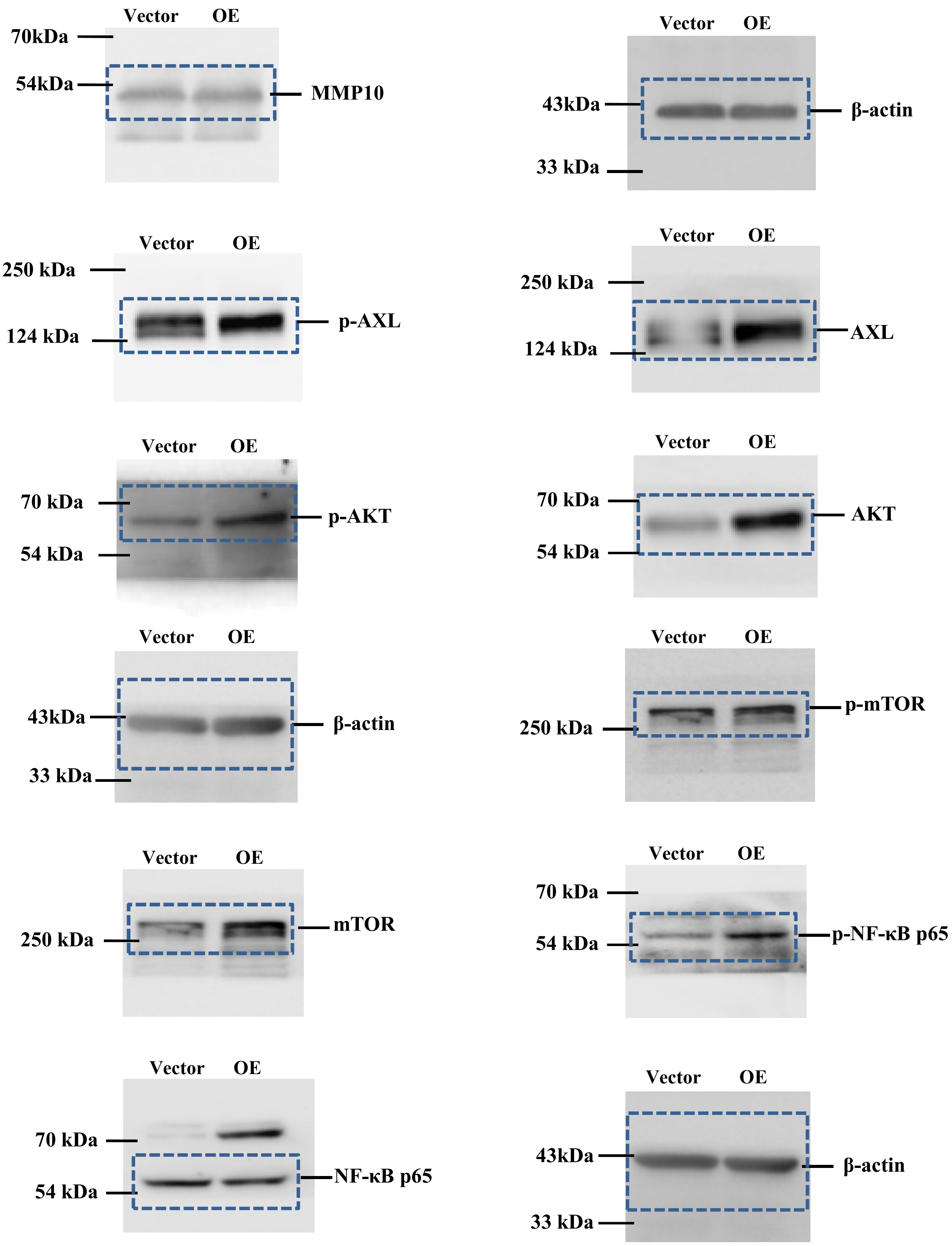

Supplementary Figure 20: Uncropped Western blots used in Figure 4b (column 2—AW8507-*MMP10* KD (sh-2)-*AXL* overexpression). Cropped sections marked on the blots are used as figures in the manuscript:

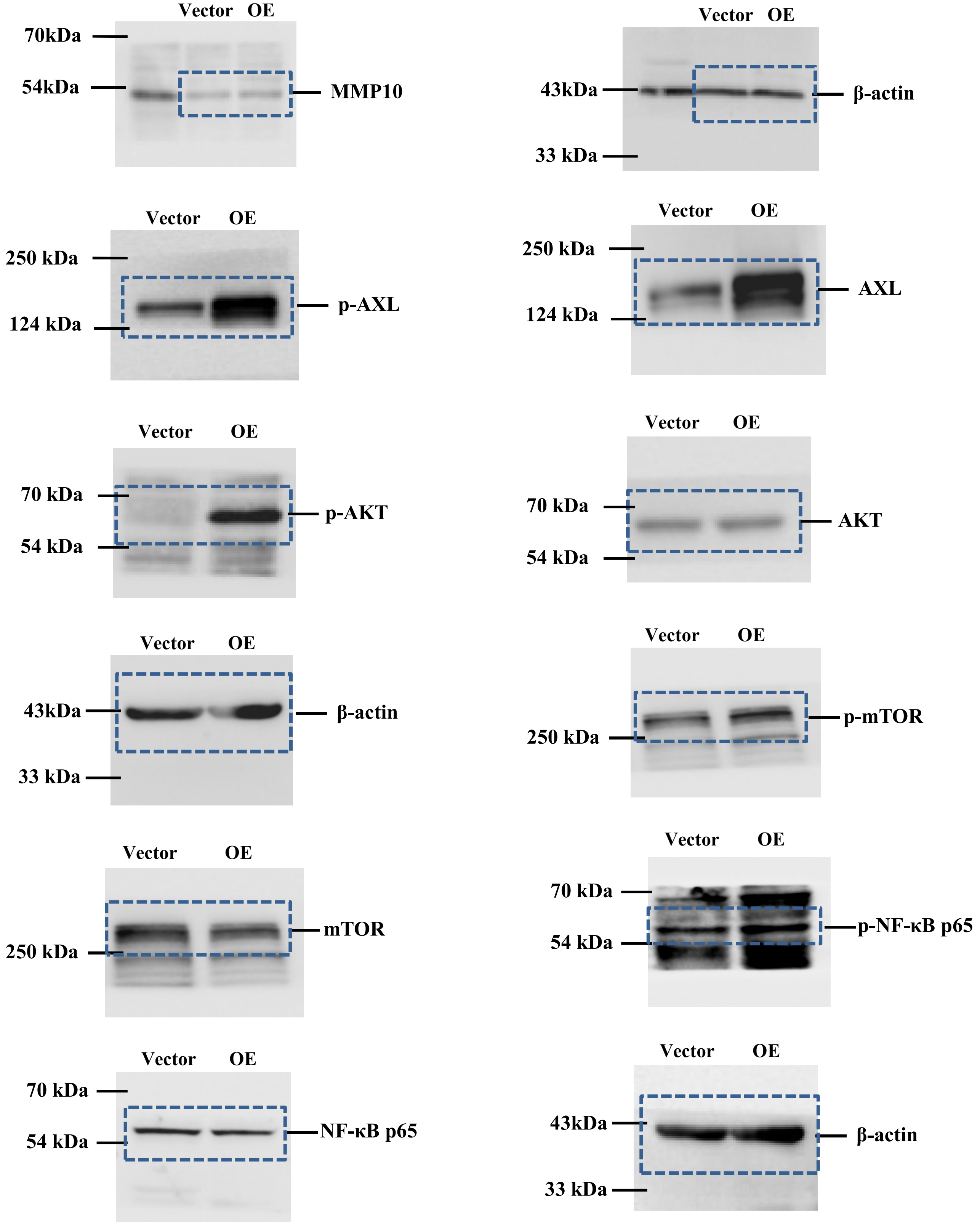

Supplementary Figure 21: Uncropped Western blots used in Figure 4b (column 3—AW13516-*MMP10* OE-*AXL* knockdown). Cropped sections marked on the blots are used as figures in the manuscript:

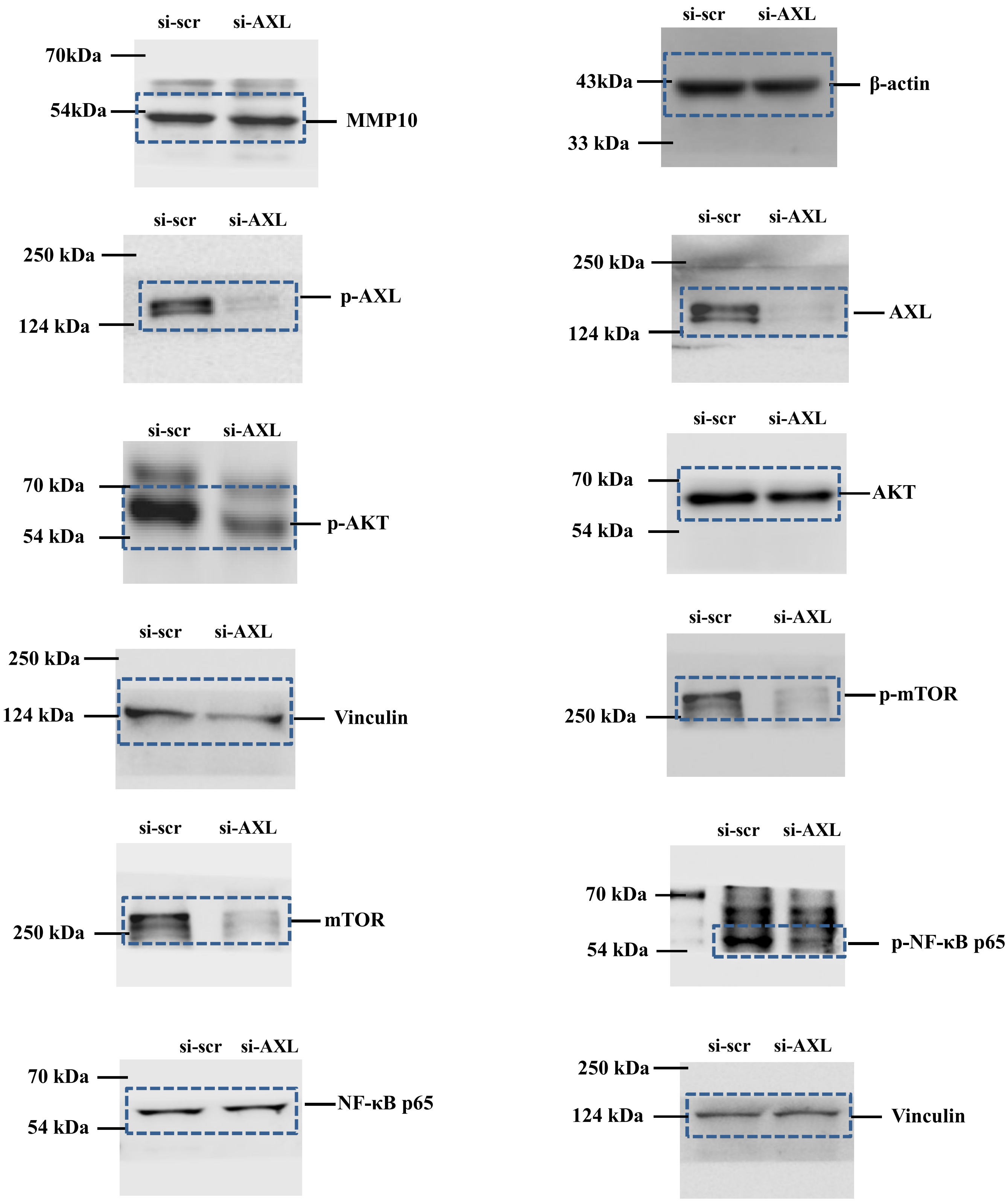

Supplementary Figure 22: Uncropped Western blots used in Supplementary Figure 2c (endogenous expression of MMP10 in tongue cancer cells). Cropped sections marked on the blots are used as figures in the manuscript:

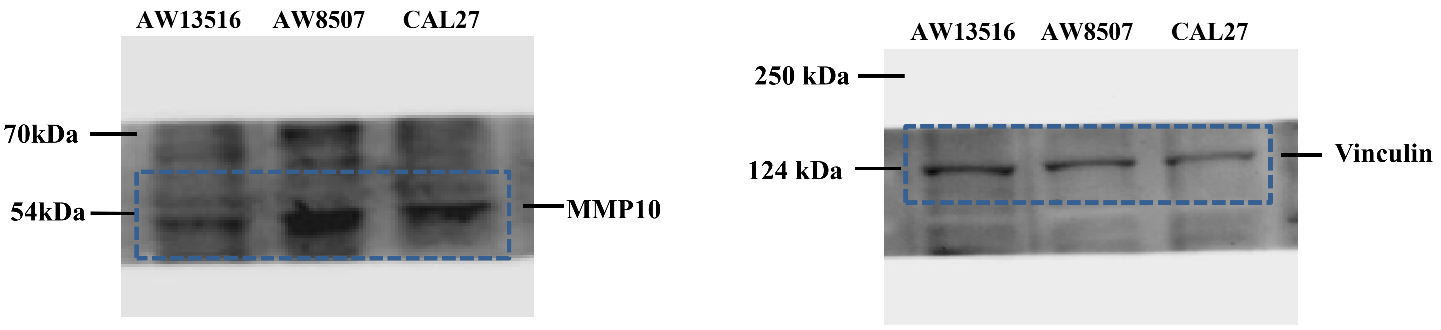

Supplementary Figure 23: Uncropped Western blots used in Supplementary Figure 3a (AW8507-*MMP10* knockdown), 3e (CAL27-*MMP10* knockdown). Cropped sections marked on the blots are used as figures in the manuscript:

Supplementary Figure 3a

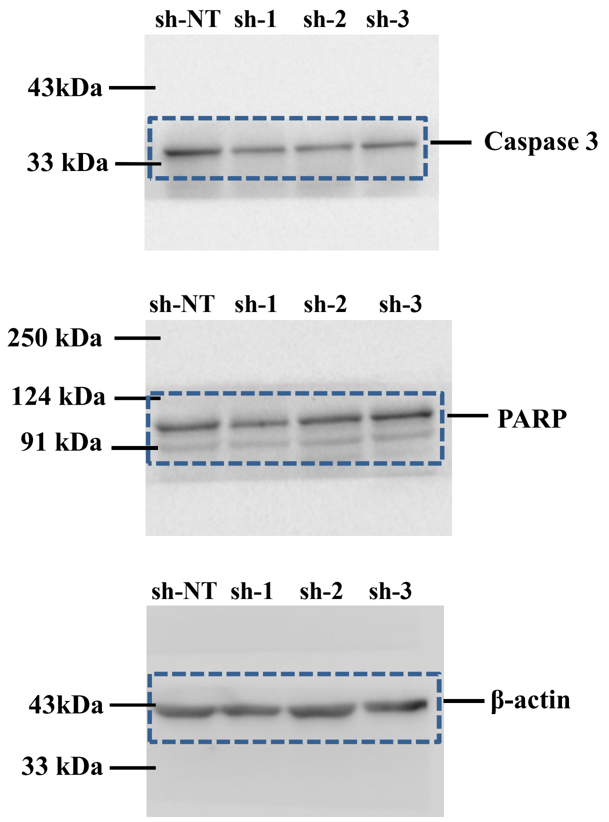

Supplementary Figure 3e

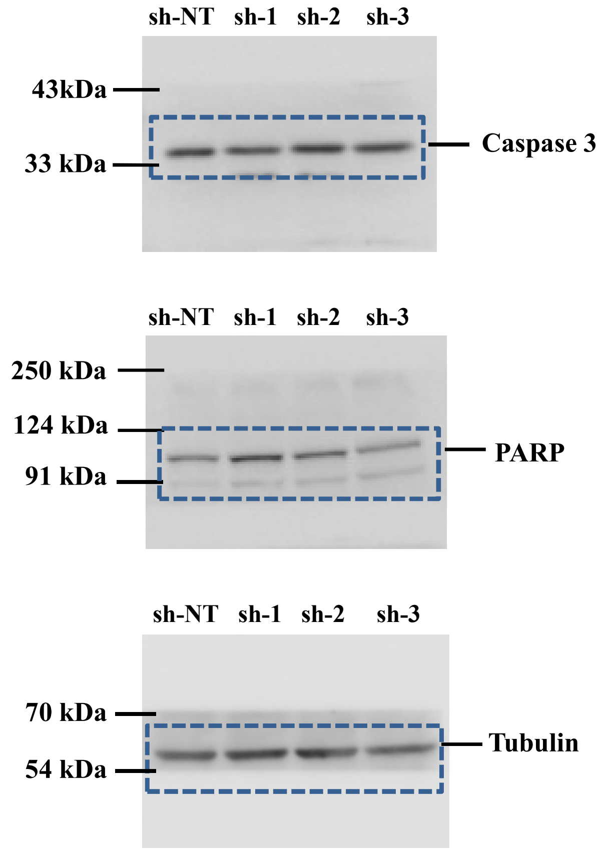

Supplementary Figure 24: Uncropped Western blots used in Supplementary Figure 7d (expression of EMT markers upon *MMP10* overexpression in AW13516 cells). Cropped sections marked on the blots are used as figures in the manuscript:

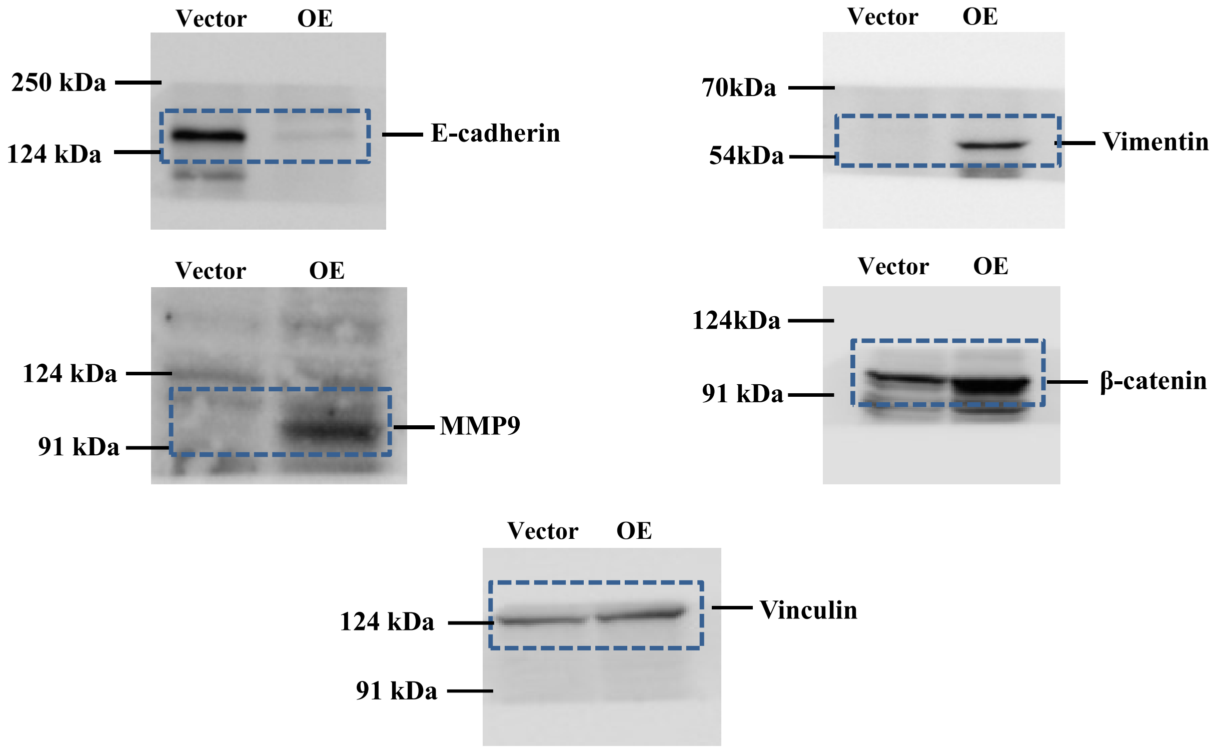

Supplementary Figure 25: Uncropped Western blots used in Supplementary Figure 10b (endogenous expression of p-AXL and AXL in tongue cancer cells). Cropped sections marked on the blots are used as figures in the manuscript:

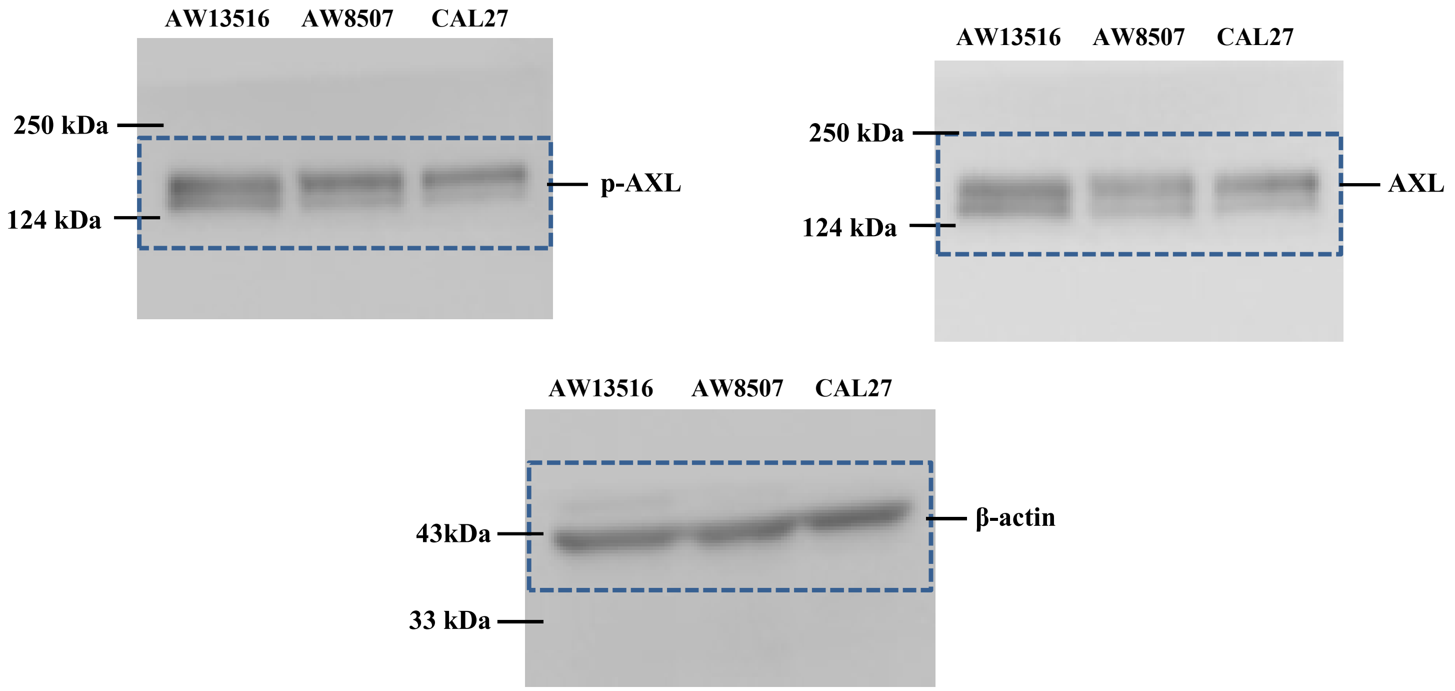

Supplementary Table 1: Association of clinical features of tongue cancer patients with MMP10 or *miR-944* expression

Clinicopathogical features	Variable	*MMP10* transcript expression (n = 110)				MMP10 protein expression (n = 98)				*miR-944* transcript expression (n = 93)			
		n (%)	High	Low	*p*-value	n (%)	High	Low	*p*-value	n (%)	Low	High	*p*-value
Age	≥65 years	15 (14%)	12	3	0.6440	7 (7%)	5	2	0.1599	11 (12%)	6	5	0.8718
	<65 years	86 (78%)	64	22		91 (93%)	40	51		77 (83%)	40	37	
	NA	9 (8%)	6	3		0 (0%)	0	0		5 (5%)	1	4	
Gender	Male	76 (69%)	57	19	0.9199	72 (73%)	33	39	0.9776	67 (72%)	33	34	0.3112
	Female	25 (23%)	19	6		26 (27%)	12	14		21 (23%)	13	8	
	NA	9 (8%)	6	3		0 (0%)	0	0		5 (5%)	1	4	
Smoking	Smoker	26 (24%)	17	9	0.1763	21 (21%)	9	12	0.7508	23 (25%)	14	9	0.3368
	Non-smoker	75 (68%)	59	16		77 (79%)	36	41		65 (70%)	32	33	
	NA	9 (8%)	6	3		0 (0%)	0	0		5 (5%)	1	4	
Alcohol	Yes	21 (19%)	11	10	0.0637	16 (16%)	9	7	0.3646	22 (24%)	13	9	0.4597
	No	80 (73%)	65	15		82 (84%)	36	46		66 (71%)	33	33	
	NA	9 (8%)	6	3		0 (0%)	0	0		5 (5%)	1	4	
Tobacco	Yes	75 (68%)	57	18	0.7660	47 (48%)	21	26	0.8134	63 (68%)	31	32	0.3606
	No	26 (24%)	19	7		51 (52%)	24	27		25 (27%)	15	10	
	NA	9 (8%)	6	3		0 (0%)	0	0		5 (5%)	1	4	
Chewer	Yes	72 (65%)	53	19	0.5482	32 (33%)	14	18	0.7642	64 (69%)	33	31	0.8276
	No	29 (26%)	23	6		66 (67%)	31	35		24 (26%)	13	11	
	NA	9 (8%)	6	3		0 (0%)	0	0		5 (5%)	1	4	

NA = Information not available

Supplementary Table 2: List of miRNAs predicted to be targeting 3'-UTR of *MMP10*

S.No.	miRNA	miRWalk	miRanda	mirbridge	miRDB	miRMap	miRNAMap	Pictar2	PITA	RNAhybrid	Targetscan	Number of algorithm predicting	Cancer type	Expression status
1	*miR-130a-3p*											7	Other cancer	up/down
2	*miR-148a-3p*											7	HNSCC	down
3	*miR-148b-3p*											7	Other cancer	down
4	*miR-152-3p*											7	Other cancer	down
5	*miR-453-3p*											7	Other cancer	up/down
6	*miR-496*											7	novel	unknown
7	*miR-944*											7	Other cancer	mixed
8	*miR-301b*											7	Other cancer	up
9	*miR-130b*											7	HNSCC	up
10	*miR-1229*											7	Other cancer	up
11	*miR-301a*											7	Other cancer	up

Supplementary Table 3: List of significantly differentially expressed miRNAs identified in node positive tumors (pT2N1) compared to node negative (pT2N0)

Upregulated (log2FC>0.5; *p*-val<0.05)		
S.No.	miRNA	log2FC
1	*miR-3124*	1.51
2	*miR-548x*	1.36
3	*miR-4654*	1.19
4	*miR-4322*	1.14
5	*miR-3180*	1.14
6	*miR-92b*	1.13
7	*miR-4443*	1.07
8	*miR-548-3p*	1.02
9	*miR-2277*	1.01
10	*miR-1909*	0.94
11	*miR-1228*	0.87
12	*miR-3669*	0.87
13	*miR-3121*	0.86
14	*miR-548c*	0.83
15	*miR-4706*	0.82
16	*miR-4704*	0.78
17	*miR-1184*	0.78
18	*miR-4723*	0.77
19	*miR-548-5p*	0.64

Downregulated (log2FC<-0.5; *p*-val<0.05)		
S.No.	miRNA	log2FC
1	*miR-4315*	-0.69
2	*miR-4772*	-0.71
3	*miR-3174*	-0.74
4	*miR-532*	-0.74
5	*miR-425*	-0.86
6	*miR-652*	-0.97
7	*miR-203*	-1.09
8	*miR-183-5p*	-1.09
9	*miR-944*	-1.40
10	*miR-4701*	-1.40
11	*miR-183-3p*	-1.56

Supplementary Table 4: List of significantly differentially expressed genes identified in AW13516 clones overexpressing *MMP10*

Upregulated (log2FC>1.5; p-val<0.05)					
S.No.	Gene name	Cuffdiff		NOISeq	
		log2FC	p-value	log2FC	prob
1	CDH11	4.80	0.0001	4.66	0.9999
2	INHBA	4.29	0.0001	3.88	0.9997
3	THBS1	3.61	0.0001	3.46	0.9990
4	CPA4	3.41	0.0001	3.38	0.9987
5	GDA	2.84	0.0001	2.95	0.9949
6	PFKFB4	3.06	0.0002	3.17	0.9935
7	FSTL1	3.23	0.0004	2.80	0.9934
8	KRT14	2.77	0.0001	2.74	0.9923
9	COL16A1	2.39	0.0061	2.76	0.9920
10	GGT4P	2.51	0.0179	2.54	0.9873
11	IL7R	2.40	0.0016	2.56	0.9866
12	IL6	2.91	0.0004	2.49	0.9859
13	KRT81	2.35	0.0001	2.40	0.9843
14	DKK1	2.50	0.0491	2.41	0.9840
15	PTGS2	2.75	0.0014	2.50	0.9837
16	ZBED2	2.37	0.0005	2.37	0.9831
17	FSTL3	2.32	0.0001	2.34	0.9831
18	SCG2	3.13	0.0053	3.12	0.9831
19	PMEPA1	2.50	0.0054	2.29	0.9819
20	COL5A1	2.19	0.0001	2.24	0.9813
21	COL1A1	2.06	0.0248	2.20	0.9805
22	IL11	2.89	0.0140	2.81	0.9803
23	DLC1	2.34	0.0005	2.20	0.9802
24	EXOC3	2.80	0.0001	2.16	0.9783
25	SHISAL1	2.19	0.0005	2.12	0.9770
26	COL12A1	2.37	0.0001	2.10	0.9766
27	TH	2.42	0.0399	4.09	0.9761
28	CXCL1	1.98	0.0003	2.08	0.9760
29	KRT17	1.74	0.0039	2.05	0.9750
30	DLX2	2.07	0.0058	2.13	0.9742
31	FGFBP1	2.01	0.0001	2.02	0.9722
32	AREG	2.11	0.0050	2.10	0.9711
33	RRAD	1.98	0.0005	1.96	0.9648
34	HMOX1	1.69	0.0119	1.95	0.9622
35	IFI6	1.85	0.0008	1.91	0.9620
36	TBX3	2.11	0.0008	1.92	0.9611
37	WNT7A	1.81	0.0003	1.81	0.9575
38	GPR39	1.92	0.0083	1.90	0.9570
39	AXL	1.79	0.0002	1.80	0.9568
40	MICAL2	1.91	0.0003	1.80	0.9567
41	MN1	1.85	0.0177	1.94	0.9565
42	KCNMA1	2.29	0.0005	1.79	0.9565
43	PAPPA	2.09	0.0270	2.11	0.9552
44	MMP2	1.75	0.0002	1.76	0.9550
45	PGBD5	2.81	0.0021	2.02	0.9545
46	GNG11	1.84	0.0082	1.82	0.9541
47	LYPD1	1.94	0.0482	1.86	0.9536
48	PCSK1N	1.67	0.0120	1.86	0.9529
49	TNFRSF12A	1.76	0.0001	1.67	0.9521
50	CAPG	1.52	0.0006	1.64	0.9515
51	ISG15	1.55	0.0007	1.63	0.9508
52	DUSP4	1.66	0.0001	1.63	0.9508

Downregulated (log2FC<-1.5; p-val<0.05)					
S.No.	Gene name	Cuffdiff		NOISeq	
		log2FC	p-value	log2FC	prob
1	SORBS1	-5.07	0.0001	-4.74	0.9999
2	KCNK3	-4.70	0.0022	-4.34	0.9992
3	CEL	-4.11	0.0011	-3.59	0.9980
4	EGLN3	-4.10	0.0080	-4.24	0.9978
5	BMP7	-3.09	0.0004	-3.27	0.9976
6	SLC6A12	-2.54	0.0043	-3.24	0.9975
7	ANGPTL4	-3.32	0.0001	-3.18	0.9973
8	KHDRBS3	-3.47	0.0002	-3.21	0.9951
9	NXPH4	-2.59	0.0007	-2.94	0.9947
10	MT-ATP8	-2.08	0.0060	-2.84	0.9942
11	SERPINA1	-2.88	0.0001	-2.78	0.9931
12	MT-ATP6	-2.08	0.0060	-2.73	0.9923
13	DNM1	-2.43	0.0020	-2.74	0.9923
14	HIST2H2BD	-3.29	0.0220	-2.86	0.9921
15	LCN2	-2.76	0.0004	-2.75	0.9915
16	AC239868.1	-2.60	0.0014	-2.66	0.9913
17	C1QL1	-2.69	0.0003	-2.62	0.9903
18	CD14	-2.40	0.0091	-2.71	0.9899
19	NOTUM	-2.18	0.0220	-2.74	0.9899
20	MT-CYB	-2.57	0.0001	-2.49	0.9860
21	MTND2P28	-3.97	0.0276	-2.43	0.9851
22	TOX2	-2.50	0.0005	-2.41	0.9845
23	PADI3	-2.49	0.0001	-2.39	0.9838
24	TJP3	-3.00	0.0472	-3.08	0.9832
25	PPFIA4	-2.93	0.0371	-3.81	0.9830
26	NCAM1	-2.52	0.0001	-2.31	0.9824
27	CLDN4	-2.26	0.0005	-2.20	0.9802
28	GAA	-2.13	0.0004	-2.14	0.9779
29	DUSP9	-2.49	0.0061	-2.59	0.9770
30	HIST2H2BE	-2.14	0.0017	-2.16	0.9770
31	AQP3	-2.06	0.0008	-2.08	0.9755
32	FXYD2	-2.48	0.0013	-2.12	0.9745
33	ASIC1	-2.59	0.0022	-2.15	0.9735
34	PCDH1	-2.02	0.0079	-2.13	0.9727
35	MTND4P12	-1.86	0.0013	-2.02	0.9722
36	ZNF774	-2.56	0.0130	-2.65	0.9657
37	EEF1A2	-2.08	0.0001	-1.96	0.9649
38	CCL5	-2.14	0.0007	-1.96	0.9644
39	AL365181.2	-2.16	0.0069	-2.17	0.9643
40	MELTF	-2.01	0.0024	-1.94	0.9630
41	NDRG1	-1.95	0.0001	-1.89	0.9613
42	ISYNA1	-1.77	0.0066	-1.88	0.9613
43	NGFR	-2.10	0.0001	-1.88	0.9613
44	FXYD6-FXYD2	-2.48	0.0013	-2.25	0.9607
45	CITED2	-2.34	0.0007	-1.87	0.9606
46	LAT2	-2.24	0.0084	-1.92	0.9588
47	MXI1	-2.22	0.0021	-1.90	0.9574
48	MIR205HG	-1.54	0.0074	-1.79	0.9565
49	SCNN1A	-2.04	0.0079	-1.77	0.9554
50	MT-CO3	-2.08	0.0060	-1.76	0.9550
51	CDK18	-1.69	0.0021	-1.73	0.9534
52	AL365181.3	-1.61	0.0059	-1.72	0.9532
53	MT-CO2	-2.08	0.0060	-1.70	0.9531
54	GLUL	-1.83	0.0071	-1.70	0.9530
55	DDIT4	-1.59	0.0034	-1.69	0.9527
56	SLC6A8	-1.73	0.0002	-1.68	0.9522
57	MT-ND4	-2.08	0.0060	-1.65	0.9516
58	REEP6	-1.81	0.0052	-1.72	0.9506

Supplementary Table 5: Reactome pathway analysis of genes upregulated upon overexpression of *MMP10* in AW13516 cell line

Pathway name	#Entities found	Entities pValue	Entities FDR	Submitted entities found
Signaling by Interleukins	13	1.20E-05	9.87E-04	DUSP4;IL11;IL6;MMP2;HMOX1;CXCL1;IL7R;PTGS2
Signaling by Receptor Tyrosine Kinases	9	0.003217561	0.069211641	DUSP4;COL1A1;FGFBP1;COL5A1;RRAD;AXL;AREG;THBS1
Interleukin-4 and Interleukin-13 signaling	8	1.00E-05	9.87E-04	IL6;MMP2;HMOX1;PTGS2
Regulation of IGF transport and uptake by IGFBPs	7	2.92E-06	5.63E-04	IL6;MMP2;PAPPA;SCG2;FSTL1;FSTL3
Interleukin-10 signaling	6	4.11E-06	5.63E-04	IL6;CXCL1;PTGS2
Extracellular matrix organization	6	0.005242896	0.094372127	COL1A1;COL16A1;COL5A1;MMP2;COL12A1;THBS1
Collagen degradation	5	2.28E-05	0.001548509	COL1A1;COL16A1;COL5A1;MMP2;COL12A1
Post-translational protein phosphorylation	5	1.93E-04	0.00868779	IL6;SCG2;FSTL1;FSTL3
Signaling by TGFB family members	5	2.37E-04	0.009723192	PMEPA1;INHBA;SCG2;FSTL1;FSTL3
Formation of the cornified envelope	5	5.64E-04	0.017481866	KRT81;KRT17;KRT14
Degradation of the extracellular matrix	5	7.71E-04	0.022358477	COL1A1;COL16A1;COL5A1;MMP2;COL12A1
Keratinization	5	0.004798611	0.091173616	KRT81;KRT17;KRT14
Anti-inflammatory response favouring Leishmania parasite infection	5	0.01448997	0.173879643	MN1;GPR39;IL6;GNG11
Leishmania parasite growth and survival	5	0.01448997	0.173879643	MN1;GPR39;IL6;GNG11
Signaling by WNT	5	0.022252494	0.210790327	TH;WNT7A;SCG2;GNG11;DKK1
Interferon Signaling	5	0.044497307	0.210790327	MN1;IFI6;ISG15
Leishmania infection	5	0.045288352	0.210790327	MN1;GPR39;IL6;GNG11
Collagen chain trimerization	4	6.61E-05	0.00383323	COL1A1;COL16A1;COL5A1;COL12A1
Collagen biosynthesis and modifying enzymes	4	5.24E-04	0.017481866	COL1A1;COL16A1;COL5A1;COL12A1
Integrin cell surface interactions	4	8.65E-04	0.023346352	COL1A1;COL16A1;COL5A1;THBS1
Collagen formation	4	0.001660709	0.041517719	COL1A1;COL16A1;COL5A1;COL12A1
ADORA2B mediated anti-inflammatory cytokines production	4	0.007447123	0.115765055	GPR39;IL6;GNG11
Interferon alpha/beta signaling	4	0.013832516	0.173879643	IFI6;ISG15

Supplementary Table 6: Primer sequences used for real-time PCR

Primer ID	Primer sequence
OAD1613_*MMP10* _FP	ATCTGAGATGCCAGCCAAGT
OAD1614_*MMP10* _RP	AGGGTTCCAGTGGGATCTTC
OAD1615_*GAPDH* _FP	AATCCCATCACCATCTTCCA
OAD1616_*GAPDH* _RP	TGGACTCCACGACGTACTCA
OAD2252_*CDH11* _FP	TCTTTGCAGCAGAAATCCAC
OAD2253_*CDH11* _RP	AATTGGCTGGTTGGAAAGTG
OAD2254_*IL6* _FP	CAAATTCGGTACATCCTCGAC
OAD2255_*IL6* _RP	GCAAGTCTCCTCATTGAATCC
OAD2258_*COL1A1* _FP	GTATTGCTGGACAGCGTGGT
OAD2259_*COL1A1* _RP	TCACCACTTGCTCCAGAG
OAD2260_*AXL* _FP	TGGCTGTGAAGACGATGAAG
OAD2261_*AXL* _RP	CTGGGAAGCTCTCTCGTTC
OAD2262_*IL11* _FP	CTGAGGGACAAATTCCCAGC
OAD2263_*IL11* _RP	GTGCCGCAGGTAGGACAGTAG
OAD2264_*IL7R* _FP	ATCGCAGCACTCACTGACCT
OAD2265_*IL7R* _RP	TCAGGCACTTTACCTCCACG
OAD1846_*MMP2* _FP	CCCTGATGTCCAGCGAGTG
OAD1847_*MMP2* _RP	ACGACGGCATCCAGGTTATC
OAD2270_*FGFBP1* _FP	GCAGATGGGCTGCTACTGAG
OAD2271_*FGFBP1* _RP	TAGGCATGAGGTTGGATTGC
OAD607_*CDH2* _FP	TCCAGACCCCAATTCAATTAATATTAC
OAD608_*CDH2* _RP	AAAATCACCATTAAGCCGAGTGA
OAD609_*CDH1* _FP	TGAGTGTCCCCCGGTATCTTC
OAD610_*CDH1* _RP	CAGTATCAGCCGCTTTCAGATTTT
SNAI2 _FP	TTTCTTGCCCTCACTGCAAC
SNAI2 _RP	ACAGCAGCCAGATTCCTCAT
OAD1481_*miR-148a* _FP	GCGCTCAGTGCACTACAGAACTTTGT
OAD1482_*miR-152* _FP	GGCTCAGTGCATGACAGAACTTGG
OAD1483_*miR-148b* _FP	GCGCTCAGTGCATCACAGAACTTTGT
OAD1484_*miR-453-3p* _FP	GCGCTAGTGCAATATTGCTTATAGGGT
OAD1485_*miR-130a* _FP	GCCAGTGCAATGTTAAAAGGGCAT
OAD1486_*miR-496* _FP	GCGCTGAGTATTACATGGCCAATCTC
OAD943_*miR-944* _FP	CCGAAATTATTGTACATCGGATGAG
U6 _FP	GCTTCGGCAGCACATATACTAAAAT
U6 _RP	CGCTTCACGAATTTGCGTGTCAT

Supplementary Table 7: Primer sequences used for cloning

Primer ID	Primer sequence
OAD983_*MMP10*_cloning_FP	CCGGGATTCATGATGCATCTTGCATTCC
OAD985_*MMP10*_cloning_RP	GGCGAATTCCTAGCAATGTAACCAGCTG
OAD971_*MMP10*_3'UTR_cloning_FP	GCTCTAGAGCGAGATAGGGGGAAGAC
OAD972_*MMP10*_3'UTR_cloning_RP	GCTCTAGAAGAAAGTAAGGAACAGGCC
OAD_1264_*miR-944*_cloning_FP	GTAGGATCCCACCAACTAACAAATTCAG
OAD_1265_*miR-944*_cloning_RP	GTACTCGAGCACTAGACAGATTCTCC

Supplementary Table 8: Primer sequences used for siRNA-mediated knockdown of *AXL* and *MMP10*

Primer ID	Primer sequence
OAD2306_T7 promoter_FP	TAATACGACTCACTATAG
OAD2346_*AXL*_as_siRNA1	AAGACATCCTCTTTCTCCTGCCTATAGTGAGTCGTATTA
OAD2347_*AXL*_s_siRNA1	TTCGCAGGAGAAAGAGGATGTCTATAGTGAGTCGTATTA
OAD2348_*AXL*_as_siRNA2	AAGATTTGGAGAACACACTGACTATAGTGAGTCGTATTA
OAD2349_*AXL*_s_siRNA2	CCTTCAGTGTGTTCTCCAAATCTATAGTGAGTCGTATTA
OAD2635_*MMP10*_s_siRNA1	ATGAAGTTAACAGCAGGGACACTATAGTGAGTCGTATTA
OAD2636_*MMP10*_as_siRNA1	CGGTGTCCCTGCTGTTAACTTCTATAGTGAGTCGTATTA
OAD2637_*MMP10*_s_siRNA2	AGGAGTTGAGCCTAAGGTTGACTATAGTGAGTCGTATTA
OAD2638_*MMP10*_as_siRNA2	GCATCAACCTTAGGCTCAACTCTATAGTGAGTCGTATTA
