# Supplementary material for: Role of miR-944/MMP10/AXL- axis in lymph node metastasis in tongue cancer
Source: Commun Biol. 2023 Jan 17;6:57. doi: 10.1038/s42003-023-04437-6 (PMC9845355; doi:10.1038/s42003-023-04437-6)
Supplement: Supplementary file 5 — Reporting Summary-New [file 42003_2023_4437_MOESM5_ESM.pdf]

nature portfolio

Corresponding author(s): Sudhir Nair, Amit Dutt

Last updated by author(s): Jan 5, 2023

Reporting Summary

Nature Portfolio wishes to improve the reproducibility of the work that we publish. This form provides structure for consistency and transparency in reporting. For further information on Nature Portfolio policies, see our Editorial Policies and the Editorial Policy Checklist.

Statistics

For all statistical analyses, confirm that the following items are present in the figure legend, table legend, main text, or Methods section.

n/a	Confirmed	
☐	☒	The exact sample size (*n*) for each experimental group/condition, given as a discrete number and unit of measurement
☐	☒	A statement on whether measurements were taken from distinct samples or whether the same sample was measured repeatedly
☐	☒	The statistical test(s) used AND whether they are one- or two-sided<br>*Only common tests should be described solely by name; describe more complex techniques in the Methods section.*
☐	☒	A description of all covariates tested
☒	☐	A description of any assumptions or corrections, such as tests of normality and adjustment for multiple comparisons
☐	☒	A full description of the statistical parameters including central tendency (e.g. means) or other basic estimates (e.g. regression coefficient) AND variation (e.g. standard deviation) or associated estimates of uncertainty (e.g. confidence intervals)
☐	☒	For null hypothesis testing, the test statistic (e.g. *F*, *t*, *r*) with confidence intervals, effect sizes, degrees of freedom and *P* value noted<br>*Give P values as exact values whenever suitable.*
☒	☐	For Bayesian analysis, information on the choice of priors and Markov chain Monte Carlo settings
☒	☐	For hierarchical and complex designs, identification of the appropriate level for tests and full reporting of outcomes
☐	☒	Estimates of effect sizes (e.g. Cohen's *d*, Pearson's *r*), indicating how they were calculated

Our web collection on statistics for biologists contains articles on many of the points above.

Software and code

Policy information about availability of computer code

Data collection	No software was used for data collection. For survival analysis, TCGA-HNSC clinical data was directly imported into the Kaplan-Meier plotter server using the Pan-cancer RNA-seq data.

Data analysis	Transcriptome sequencing data analysis was performed using the Tuxedo-suite pipeline. In brief, alignment of short reads was done against the reference genome (GRCh38) using TopHat2. Cufflinks v.2.0.2 was used to find the expressed transcripts in the data and cuffdiff was used for the identification of differentially expressed genes. Also, NOISeq was used for differential expression analysis and overlapping genes were prioritized for functional experiments. Differentially expressed genes were used for the identification of enriched or depleted pathways by Reactome pathway analysis tool.<br><br>miRNA microarray data (.cel files) were pre-processed using RMA method which includes background correction, log transformation, quantile normalization followed by essential quality control steps. Probe-centric data were converted into miRNA-centric data using an average over-replicate approach. We excluded snoRNA, hypothetical miRNA, control probes from the analysis and restricted analysis only for annotated microRNAs (miRBase-20 release), and the data was analyzed using BRB-ArrayTools developed by Dr. Richard Simon and the BRB-ArrayTools Development Team.<br><br>Survival analysis was performed using Kaplan-Meier plotter online tool in 499 TCGA-HNSC samples. TCGA-HNSC clinical data was directly imported into the Kaplan-Meier plotter server using the Pan-cancer RNA-seq data.<br><br>Correlation between expression of genes and nodal status/ other clinical parameters, in the in-house and TCGA-HNSC data, was performed using R programming (www.r-project.org). Statistical analysis was performed using GraphPad Prism version 8 software (GraphPad Software, La Jolla, CA). The student's unpaired t-test was used to determine the statistical significance between different groups.

For manuscripts utilizing custom algorithms or software that are central to the research but not yet described in published literature, software must be made available to editors and reviewers. We strongly encourage code deposition in a community repository (e.g. GitHub). See the Nature Portfolio guidelines for submitting code & software for further information.

Data

Policy information about availability of data

All manuscripts must include a data availability statement. This statement should provide the following information, where applicable:

- Accession codes, unique identifiers, or web links for publicly available datasets
- A description of any restrictions on data availability
- For clinical datasets or third party data, please ensure that the statement adheres to our policy

The transcriptome sequencing data generated and analysed during the current study is available in the ArrayExpress repository under the accession number: E-MTAB-11185. The source data behind all the graphs in the paper are provided in the Supplementary Data 1 and the uncropped raw western blot images (Supplementary Fig. 14-25) are provided in the Supplementary Information.

Human research participants

Policy information about studies involving human research participants and Sex and Gender in Research.

Reporting on sex and gender	Gender (self reported) was considered in the study design. The findings of the study applies to both the genders. The consent was obtained from all the patients for using the gender information data. We have performed gender-based analyses to study if MMP10 or miR-944 expression are associated with any specific gender. However, we did not find any significant results in the study based on these comparisons.
Population characteristics	Please refer to Table 2 of the manuscript.
Recruitment	A total of 130 fresh frozen tongue tumor samples and 98 formalin-fixed paraffin-embedded (FFPE) tissue blocks with adequate tumor content and quality were collected from the tumor tissue repository of Tata Memorial Hospital (TMH-TTR) and the Advanced Centre for Treatment, Research and Education in Cancer (ACTREC-TTR).
Ethics oversight	Samples were collected with the approval of The Institutional Review Board (IRB) and the Ethics Committee (EC) of Tata Memorial Centre-ACTREC.

Note that full information on the approval of the study protocol must also be provided in the manuscript.

Field-specific reporting

Please select the one below that is the best fit for your research. If you are not sure, read the appropriate sections before making your selection.

☒ Life sciences ☐ Behavioural & social sciences ☐ Ecological, evolutionary & environmental sciences

For a reference copy of the document with all sections, see nature.com/documents/nr-reporting-summary-flat.pdf

Life sciences study design

All studies must disclose on these points even when the disclosure is negative.

Sample size	The sample size used in the study was not predetermined. 130 fresh frozen tongue tumor samples and 98 formalin-fixed paraffin-embedded (FFPE) tissue blocks were collected from the tumor tissue repository of Tata Memorial Hospital and TMC-ACTREC.
Data exclusions	No data was excluded from the analyses.
Replication	Reproducibility of the experimental findings were confirmed by performing at least 3 biological replicates of each experiment. The findings of all the biological replicates were consistent.
Randomization	Randomization was not required for our study as we screened for the expression of MMP10 in pathologically classified node positive and node negative tongue tumor samples. For in vivo studies, mice were randomized before injection of the cells orthotopically in the tongue.
Blinding	Investigators were blinded to sample identity during immunohistochemistry of tumor sections. Immunohistochemical staining was evaluated blindly by two independent pathologists.

Reporting for specific materials, systems and methods

We require information from authors about some types of materials, experimental systems and methods used in many studies. Here, indicate whether each material, system or method listed is relevant to your study. If you are not sure if a list item applies to your research, read the appropriate section before selecting a response.

Materials & experimental systems

n/a	Involved in the study
☐	☒ Antibodies
☐	☒ Eukaryotic cell lines
☒	☐ Palaeontology and archaeology
☐	☒ Animals and other organisms
☒	☐ Clinical data
☒	☐ Dual use research of concern

Methods

n/a	Involved in the study
☒	☐ ChIP-seq
☐	☒ Flow cytometry
☒	☐ MRI-based neuroimaging

Antibodies

Antibodies used

MMP10 (Cat No. MAB910, Bi Biotech)
phospho-AXL (Cat No. DY702, Cell Signaling Technology)
AXL (Cat No. C89E7, Cell Signaling Technology)
E-cadherin (Cat No. 3195T, Cell Signaling Technology)
Vimentin (Cat No. 5741T, Cell Signaling Technology)
MMP9 (Cat No. 13667T, Cell Signaling Technology)
β-catenin (Cat No. sc-7963, Santa Cruz Biotechnology)
phospho-mTOR (Cat No.5536T, Cell Signaling Technology)
mTOR (Cat No.2983T, Cell Signaling Technology)
phospho-AKT (Cat No. 4060T, Cell Signaling Technology)
AKT (Cat No. 4685S, Cell Signaling Technology)
phospho-NF-κB (Cat No.3033T, Cell Signaling Technology)
NF-κB (Cat No. ab16502, abcam)
Vinculin (Cat No. 4650S, Cell Signaling Technology)
GAPDH (Cat No. sc-32233, Santa Cruz Biotechnology)
β-actin (Cat No. sc-47778, Santa Cruz Biotechnology)
Caspase 3 (Cat No. 9662S, Cell Signaling Technology)
PARP (Cat No. 9542S, Cell Signaling Technology)
α-Tubulin (Cat No. T5168, Sigma-Aldrich)
Goat anti-rabbit IgG-HRP secondary antibody (sc-2004, Santa Cruz Biotechnology)
Goat anti-mouse IgG-HRP secondary antibody (sc-2005, Santa Cruz Biotechnology)

Validation

All the antibodies were commercially purchased and were previously validated by the vendors and described in literature. In detail:
MMP10 (Cat No. MAB910, Bi Biotech)- Immunogen Mouse myeloma cell line NS0--derived recombinant human MMP-10 Tyr18-Cys476; Applications- WB, IHC
phospho-AXL (Cat No. DY702, Cell Signaling Technology)-Phospho-Axl (Tyr702) Rabbit mAb detects endogenous levels of Axl only when phosphorylated at Tyr702. Applications: WB.
AXL (Cat No. C89E7, Cell Signaling Technology)-Axl (C89E7) Rabbit mAb detects endogenous levels of total Axl protein and does not cross-react with Tyro3. Applications: WB, IP, IHC-P, IF-IC, F
E-cadherin (Cat No. 3195T, Cell Signaling Technology)- E-Cadherin (24E10) Rabbit mAb detects endogenous levels of total E-cadherin

protein. The antibody does not cross-react with related family members, such as N-cadherin. Applications: WB, IHC-P, IF-IC, F
N-cadherin (Cat No. 13116T, Cell Signaling Technology)- N-Cadherin Rabbit mAb recognizes endogenous levels of total N-cadherin protein. Some non-specific staining has been observed in mouse kidney tissue. Applications: WB, IP, IHC-Bond, IHC-P, IF-IC
Vimentin (Cat No. 5741T, Cell Signaling Technology)- Vimentin Rabbit mAb detects endogenous levels of total vimentin protein. Applications: WB, IHC-P, IF-IC, F
MMP9 (Cat No. 13667T, Cell Signaling Technology)- Monoclonal antibody is produced by immunizing animals with a synthetic peptide corresponding to residues surrounding Phe542 of human MMP-9 protein. Applications:WB, IHC-P, F
β-catenin (Cat No. sc-7963, Santa Cruz Biotechnology)- β-catenin (E-5) is a mouse monoclonal antibody raised against amino acids 680-781 mapping at the C-terminus of β-catenin of human origin. Applications: WB, IHC-P, IF, IP
phospho-mTOR (Cat No.5536T, Cell Signaling Technology)- Antigen mTOR (Ser2448) phosphate. Applications: WB, IP, IF-IC
mTOR (Cat No.2983T, Cell Signaling Technology)- mTOR (7C10) Rabbit mAb detects endogenous levels of total mTOR protein. Applications:WB, IP, IHC-P, IF-IC, F
phospho-AKT (Cat No. 4060T, Cell Signaling Technology)-Phospho-Akt (Ser473) Rabbit mAb detects endogenous levels of Akt1 only when phosphorylated at Ser473. This antibody also recognizes Akt2 and Akt3 when phosphorylated at the corresponding residues. Applications:WB, IP, IHC-P, IF-IC, F
AKT (Cat No. 4685S, Cell Signaling Technology)- Akt (pan) (11E7) Rabbit mAb detects endogenous levels of total Akt protein. This antibody does not cross-react with other related proteins. Applications:WB, IP, IHC-P, IF-IC, F
phospho-NF-κB (Cat No.3033T, Cell Signaling Technology)-Phospho-NF-kappaB p65 (Ser536) (93H1) Rabbit mAb detects NF-κB p65 only when phosphorylated at Ser536. It does not cross-react with the p50 subunit or other related proteins. Applications:WB, IP, IF-IC, F
NF-κB (Cat No. ab16502, abcam)-Synthetic peptide corresponding to Human NF-kB p65 aa 500 to the C-terminus (C terminal) conjugated to keyhole limpet haemocyanin. Applications: IHC-P,WB,IP, ICC/IF
Vinculin (Cat No. 4650S, Cell Signaling Technology)-Vinculin Antibody detects endogenous levels of total vinculin protein. This antibody also reacts with metavinculin, a 145 kDa splice variant of vinculin. Applications: WB
GAPDH (sc-32233, Santa Cruz Biotechnology)- GAPDH (6C5) is a mouse monoclonal antibody raised against GAPDH purified from muscle of rabbit origin. Applications: WB, IP, IF
β-actin (Cat No. sc-47778, Santa Cruz Biotechnology)- Anti-β-Actin Antibody (C4) is a mouse monoclonal IgG1 κ β-Actin antibody. Applications: WB, IP
Caspase 3 (Cat No. 9662S, Cell Signaling Technology)-Caspase-3 Antibody detects endogenous levels of full length caspase-3 (35 kDa) and the large fragment of caspase-3 resulting from cleavage (17 kDa). Applications: WB, IP, IHC
PARP (Cat No. 9542S, Cell Signaling Technology)-PARP Antibody detects endogenous levels of full length PARP1 (116 kDa), as well as the large fragment (89 kDa) of PARP1 resulting from caspase cleavage. The antibody does not cross-react with related proteins or other PARP isoforms. Applications: WB.
α-Tubulin (Cat No. T5168, Sigma-Aldrich)-Monoclonal Anti-a-Tubulin recognizes an epitope located in the C-terminal end of the a-tubulin isoform in a variety of organisms (e.g., human, sea urchin, Chlamydomonas). Applications: WB, IHC.
Goat anti-rabbit IgG-HRP secondary antibody (sc-2004, Santa Cruz Biotechnology)- goat anti-rabbit IgG-HRP is an affinity purified secondary antibody raised in goat against whole rabbit IgG and conjugated to HRP (horseradish peroxidase). Applications: goat anti-rabbit IgG-HRP is recommended for detection of rabbit IgG by Western Blotting.
Goat anti-mouse IgG-HRP secondary antibody (sc-2005, Santa Cruz Biotechnology)- goat anti-mouse IgG-HRP is an affinity purified secondary antibody raised in goat against mouse IgG and conjugated to HRP (horseradish peroxidase). Applications- goat anti-mouse IgG-HRP is recommended for detection of mouse IgG by Western Blotting.

Eukaryotic cell lines

Policy information about cell lines and Sex and Gender in Research

Cell line source(s)	AW13516 and AW8507 cell lines were established within Tata Memorial Centre (Tatake et al., J. Cancer Res. Clin. Oncol., 1990) and were acquired from Tata Memorial Hospital. CAL27 (CRL-2095) cell line was purchased from ATCC. Stable MMP10 overexpression or shRNA-mediated stable knockdown clones were generated in-house from parental cell lines.
Authentication	The cell lines were authenticated by DNA short tandem repeat (STR) profiling using Promega Geneprint 10 system in conjugation with GeneMarker HID software tool.
Mycoplasma contamination	Cells were tested for mycoplasma and were made mycoplasma-free using the EZKill mycoplasma removal reagent (Cat No. CCK006-1, HiMedia).
Commonly misidentified lines (See ICLAC register)	No commonly misidentified cell lines were used in the study.

Animals and other research organisms

Policy information about studies involving animals; ARRIVE guidelines recommended for reporting animal research, and Sex and Gender in Research

Laboratory animals	6-8 week old female Nude mice used in the study were obtained from TMC-ACTREC animal facility.
Wild animals	Study did not involve wild animals.
Reporting on sex	There was no sex or gender specific analyses performed in the animal studies.
Field-collected samples	Study did not involve samples collected from the field.
Ethics oversight	All in vivo experiments were performed as approved by Institutional Animal Ethics Committee (IAEC), TMC-ACTREC.

Note that full information on the approval of the study protocol must also be provided in the manuscript.

Flow Cytometry

Plots

Confirm that:

☒ The axis labels state the marker and fluorochrome used (e.g. CD4-FITC).

☒ The axis scales are clearly visible. Include numbers along axes only for bottom left plot of group (a 'group' is an analysis of identical markers).

☒ All plots are contour plots with outliers or pseudocolor plots.

☒ A numerical value for number of cells or percentage (with statistics) is provided.

Methodology

Sample preparation	AW8507- and CAL27-MMP10 knockdown clones along with scrambled control cells (0.3 million cells per clone) were re-suspended in 100 µl 1X Annexin V binding buffer and incubated for 15 min with 2 µl Annexin V-FITC conjugate on ice. The suspension was diluted to a final volume of 300 µl using ice-cold 1X Annexin V binding buffer and added 1 µl propidium iodide (PI) just before acquiring on AttuneNxT flow cytometer.
Instrument	Data were acquired with AttuneNxT Aoustic Focusing Cytometer using AttuneNxT software (Life Technologies, ThermoFisher Scientific).
Software	Data were analyzed with FlowJo v10.6.1 software.
Cell population abundance	20,000 cells for each cell population were identified using the Annexin V-FITC/PI markers and assessed the intensity. The purity of the samples was achieved by using only single and live cells for analysis (doublets and clumps of cells were excluded).
Gating strategy	Cells were gated based on FSC (size) and SSC (granularity). Further, the fluorescence was measured by Annexin V-FITC and propidium iodide (PI) using respective emission wavelength. Annexin V only staining indicate early apoptotic cells and PI only staining indicate necrotic cells, whereas Annexin V and PI double staining indicate late apoptotic cells. The background signal was determined and subtracted using unstained cells in each group.

☒ Tick this box to confirm that a figure exemplifying the gating strategy is provided in the Supplementary Information.
